# Molecular evolution between chemistry and biology: The interplay of competition, cooperation, and mutation

**DOI:** 10.1007/s00249-018-1281-7

**Published:** 2018-03-02

**Authors:** Peter Schuster

**Affiliations:** Institut für Theoretische Chemie, Universität Wien, Währingerstraße 17, 1090 Wien, Austria

**Keywords:** Competition, Cooperation, Darwinian optimization, Error threshold, Mutation, Neutral evolution, Selection

## Abstract

Biological evolution is reduced to three fundamental processes in the spirit of a minimal model: (i) Competition caused by differential fitness, (ii) cooperation of competitors in the sense of symbiosis, and (iii) variation introduced by mutation understood as error-prone reproduction. The three combinations of two fundamental processes each, (${\mathcal A}$) competition and mutation, (${\mathcal B}$) cooperation and competition, and (${\mathcal C}$) cooperation and mutation, are analyzed. Changes in population dynamics that are induced by bifurcations and threshold phenomena are discussed.

## Introduction

Biology is centered around evolutionary thinking or understanding biology implies understanding evolution as Theodosius Dobzhansky pointed out clearly in his famous book on evolution: “Nothing in biology makes sense except in the light of evolution” (Dobzhansky et al. 1977). Pure Darwinian evolution is a simple process but its embedding in nature renders it complex: Natural selection would follow uncomplicated laws in a simple environment. In the light of current molecular biology, there is need for a simple but comprehensive mathematical model of evolution to be able to account for modern genetics. The various epigenetic mechanisms have to be part of any comprehensive model of evolution and to keep such a model amenable to analysis and handling, molecular details must be reduced to a coarse-grained level. This article deals with a flexible model of evolution under defined environmental conditions. We present a concise and comprehensive review of work that was published elsewhere (Schuster 2016a, d, 2017a) together with a few new results.

The model focusses on three basic processes: (i) fitness-driven competition through differential reproductive success, (ii) reproduction-relevant cooperation between competitors, and (iii) reproduction-induced variation. The model is conceived with a modular structure and allows for the implementation of different, more or less complicated mechanisms for the processes (i), (ii), and (iii). For example, variation may be implemented by mutation, by recombination or by both. Here, we shall apply the simplest conceivable mechanisms: reproduction as single enzyme mediated replication (Biebricher 1983), cooperation of competitors as hypercycle dynamics (Eigen and Schuster 1978a, b), and variation as single point mutations based on the uniform error rate assumption (Swetina 1982).

Usage of the notion evolution is often ambiguous and a precise definition is desirable. Here evolution is understood as a process based on reproduction of a genotype being a DNA or an RNA sequence that carries encoded information on the formation of a phenotype, which is evaluated with respect to success in reproduction. The evolutionary process is built upon two foundations: (i) the dynamics at the population level and (ii) the environment dependent encoding of phenotypes in genotype. At the molecular level, the latter boils down to sequence–structure–function relations (Schuster 2016). The simplest systems that are capable of evolution in the sense of the given definition are nothing but special autocatalytic reactions involving polynucleotides under suitable conditions.

In the next "Preliminaries", some prerequisites are presented for the model, which is introduced in "A minimal mathematical model for the evolution of molecules". The model comes in a deterministic version based on kinetic differential equations or it is formulated as a stochastic process modeled by means of chemical master equations (Schuster 2016). Although almost no analytic solutions are available for master equations derived from nonlinear reaction kinetics^1^ in two or more variables, the stochastic version of the model can be studied by efficient simulation methods for the calculations of trajectories (Gillespie 1977, 2007). In three sections, solution curves for the three two-dimensional subspaces, (${\mathcal A}$) reproduction and mutation, (${\mathcal B}$) reproduction & cooperation, and (${\mathcal C}$) cooperation & mutation, are presented, analyzed and interpreted. Then follows a brief discussion of results obtained with the full three-dimensional model, reproduction & cooperation & mutation and in the final section we discuss the simple model in the context of the complex processes in nature.

## Preliminaries

Darwinian evolution is often—and incorrectly—seen as a synonym for optimization mainly because Ronald Fisher’s fundamental theorem of natural selection (Fisher 1930, 1958; Price 1972; Ewens and Lessard 2015) focusses on non-decreasing mean fitness $\phi (t)$ in evolving populations. In the simplest form derived from idealized models, the time derivative of the mean fitness is the variance of the fitness values and therefore a non-negative quantity. In reality, mean fitness is the result of many factors and as Fisher was been certainly aware, only a few cases—like single locus genetics—fulfil the theorem in pure form (for elaborate discussions see the more recent literature Plutynski 2006; Okasha 2008).

Evolution is intimately related to the environment in which it takes place and accordingly environment and environmental changes are major factors shaping evolutionary processes. Here, we are primarily interested in the internal dynamics and hence a well-defined and controllable environment is required. For this goal, we introduce a flow reactor in "Idealized environments", which represents a simple device that is not only useful for performing evolution experiments but also provides at the same time a suitable setup for theoretical modeling. More complex environments can be implemented as long as they can be cast in kinetic equations. In a separate "Basic processes", the processes that will be used to model evolution are introduced. The following section describes the deterministic and stochastic methods, which are applied to find solutions of the kinetic equations. Finally, we review some fundamental features of autocatalysis since this is the chemical counterpart of reproduction.

**Fig. 1:**
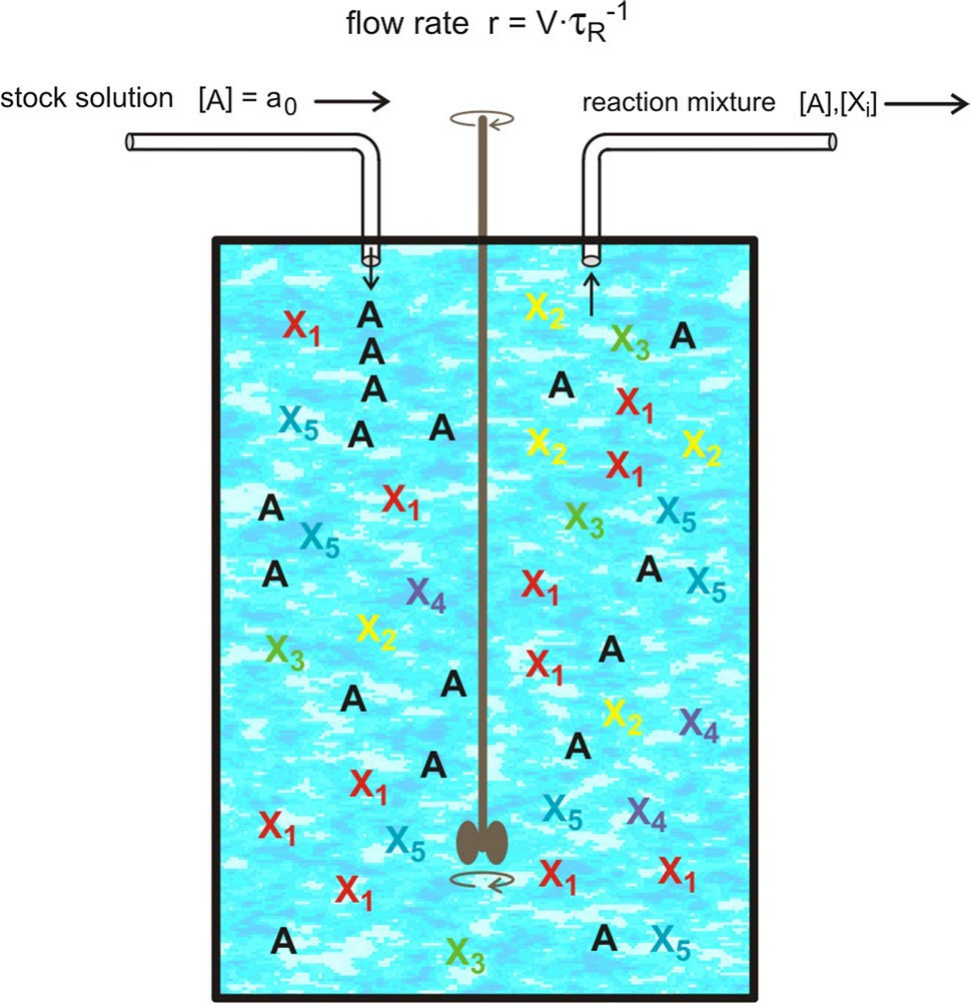
The continuous-flow stirred-tank reactor (CSTR). The figure sketches a device for controlling the environmental condition of evolution experiments. The material needed for reproduction is subsumed by **A**, it flows into the reactor with a (volumetric) flow rate *r*  [*V* / *t*]$\,\equiv \,$[volume / time] in form of a solution with concentration $[{{\mathsf{\mathbf A}}}]=a_0$ or number density $[{{\mathsf{\mathbf A}}}]=A_0$. In the reactor molecules ${{\mathsf{\mathbf X}}}_i\ (i=1,\dots ,n)$ are reproduced and **A** is consumed. The volume *V* of the reactor is constant and hence reaction mixture compensating the volume increase through influx of stock solution has to flow out of the reactor. The mean residence time of a volume element in the CSTR is $\tau _R=V/r$ [*t*]. Inflow and outflow of materials are handled as virtual chemical reactions or pseudoreactions: $*\mathop {{\longrightarrow }}\limits ^{a_0\cdot r}{{\mathsf{\mathbf A}}}$ for inflow and, ${{\mathsf{\mathbf A}}}\mathop {{\longrightarrow }}\limits ^{r}\varnothing$ and ${{\mathsf{\mathbf X}}}_i\mathop {{\longrightarrow }}\limits ^{r}\varnothing$ $(i=1,\dots ,n)$ for outflow

### Idealized environments

Environments that allow for investigations of observations as functions of one or few parameters with everything else being constant require elaborate design in the form of sophisticated experiments since devices controlling environmental conditions may be quite involved. In theoretical approaches, often the silent assumption is made that there exists a hypothetical machinery, which takes care of fixing parameter values as needed for the mathematical analysis.

Environmental influences on phenotypes are commonly large, manifold, and easy to observe. In this study, however, we are interested in the intrinsic driving forces of evolution, which result from reproduction, symbiotic cooperation and variation, and therefore impacts on evolution caused by changes in the environment are intended to be kept to a minimum. To reduce the influence of the environment as much as possible we shall assume a control device in the form of a simple flow reactor (Fig. 1; see also Schmidt 2005). More elaborate reactors, which keep, for example, the numbers of bacterial cells constant have been designed and implemented (Novick and Szillard 1950a, b; Bryson 1952). The flow reactors called chemostat, *cellstat* or turbidostat and other experimental devices for monitoring and controlling evolution in the laboratory may serve as examples (Husimi et al. 1982; Dykhuizen and Hartl 1983; Husimi 1989; Koltermann and Kettling 1997; Strunk and Ederhof 1997).

Implementation of a physical device rather than application of idealized assumptions like constant population size is required for the stochastic description of evolution. An illustrative example where the deterministic approach yields a stable solution whereas the corresponding stochastic system is unstable is provided by the linear birth-and-death process (Goel and Richter-Dyn 1974), which is described by the kinetic equations ${{\mathsf{\mathbf A}}}+{{\mathsf{\mathbf X}}}\mathop {{\longrightarrow }}\limits ^{f}2{{\mathsf{\mathbf X}}}$ and ${{\mathsf{\mathbf X}}}\mathop {{\longrightarrow }}\limits ^{d}\varnothing$. For equal birth and death rate parameters, $f=d$, the population number of the deterministic system stays constant whereas in the stochastic model the fluctuations are unregulated. With increasing amplitude of fluctuations, the populations will hit the death state, which is an absorbing boundary and where the system therefore remains caught forever. In other words, the system is unstable despite a (marginally) stable deterministic solution. The direct incorporation of constant population size into Fisher’s selection (1930) or Eigen’s equation (1971) leads to an instability of the same kind since fluctuations are not self-regulating (Jones and Leung 1981). Implementation of the system in the flow reactor provides stability due to balance control of inflow and outflow modeled by the pseudoreactions $*\mathop {{\longrightarrow }}\limits ^{a_0\cdot r}{{\mathsf{\mathbf A}}}$ and ${{\mathsf{\mathbf A}}}\mathop {{\longrightarrow }}\limits ^{r}\varnothing$ as well as ${{\mathsf{\mathbf X}}}_i\mathop {{\longrightarrow }}\limits ^{r}\varnothing$ $(i=1,\dots ,n)$ (Fig. 1).

### Basic processes

The minimal system for modeling evolution of molecules is based on the three classes of processes: (i) competitive reproduction, (ii) symbiotic cooperation, and (iii) reproduction based variation. For the minimal model we shall choose the simplest possible chemical reactions. Reproduction will be modeled by simple replication, ${{\mathsf{\mathbf A}}}+{{\mathsf{\mathbf X}}}_i\mathop {{\longrightarrow }}\limits ^{f_i}2{{\mathsf{\mathbf X}}}_i$ with $f_i$ being the fitness of species ${{\mathsf{\mathbf X}}}_i$ and competition being introduced through living on the same resource **A**. Symbiontic cooperation is introduced as catalyzed replication, ${{\mathsf{\mathbf A}}}+{{\mathsf{\mathbf X}}}_i+{{\mathsf{\mathbf X}}}_j\mathop {{\longrightarrow }}\limits ^{h_{ij}}2{{\mathsf{\mathbf X}}}_i+{{\mathsf{\mathbf X}}}_j$ $(i,j=1,\dots ,n)$, where ${{\mathsf{\mathbf X}}}_i$ represents the template and ${{\mathsf{\mathbf X}}}_j$ is the catalyst. In its most general form—every molecule ${{\mathsf{\mathbf X}}}_j$ has the potential to act as catalyst in the replication of every molecular species ${{\mathsf{\mathbf X}}}_i$—the number of catalytic terms is $n^2$ and unrealistically large since specific catalysis is a rare property. The simplest example of stable cooperative catalytic networks with fewer catalytic reactions known so far is the catalytic hypercycle (Eigen 1971; Eigen and Schuster 1977, 1978, 1978): The catalyzed reactions $2{{\mathsf{\mathbf X}}}_i+{{\mathsf{\mathbf X}}}_{i+1}\mathop {{\longleftarrow }}\limits ^{h_i}{{\mathsf{\mathbf A}}}+{{\mathsf{\mathbf X}}}_i+{{\mathsf{\mathbf X}}}_{i+1}$ ($i=1,\dots ,n;\,i\!\!\mod n$)

$$\begin{aligned} \dots \leftarrow {{\mathsf{\mathbf X}}}_n\leftarrow {{\mathsf{\mathbf X}}}_1\leftarrow {{\mathsf{\mathbf X}}}_2\leftarrow \dots \leftarrow {{\mathsf{\mathbf X}}}_{n-1}\leftarrow {{\mathsf{\mathbf X}}}_n\leftarrow \dots \end{aligned}$$

form a closed cycle with *n* members.

Genetic variation occurs at the level of a DNA or RNA genotype in forms of mutation and recombination. The simplest form of variation is the point mutation that consists of the exchange of a single nucleotide in the sequence and caused by the incorporation of a wrong nucleotide during the replication process. Correct and error-prone replication are considered as parallel reaction channels within one and the same replication mechanism (Eigen 1971). In terms of a simple over-all replication kinetics of the multistep process,

1 $$\begin{aligned} {{\mathsf{\mathbf A}}}+{{\mathsf{\mathbf X}}}_i+{{\mathsf{\mathbf E}}}\mathop {\rightarrow \rightarrow \rightarrow }\limits ^{Q_{ji}\cdot k_i[{{\mathsf{\mathbf A}}}]}{{\mathsf{\mathbf X}}}_j+{{\mathsf{\mathbf X}}}_i+{{\mathsf{\mathbf E}}}\ , \end{aligned}$$

with **E** being a replicase enzyme, the reaction rate is obtained as the product of two parameters: $Q_{ji}\cdot f_i$ where $f_i=k_i\,[{{\mathsf{\mathbf A}}}]$ is the fitness of the template that depends on the availability of resources here the concentration [ **A**]. The dimensionless factors $Q_{ji}$ with $i,j=1,\dots ,n$ are understood as the elements of a mutation matrix $\mathrm {Q}=\{Q_{ij}\}$ and provide the probability to obtain ${{\mathsf{\mathbf X}}}_j$ as a copy of the template ${{\mathsf{\mathbf X}}}_i$ that can be either correct (${{\mathsf{\mathbf X}}}_j\equiv {{\mathsf{\mathbf X}}}_i$) or error-prone (${{\mathsf{\mathbf X}}}_j$, $j\ne i$). Conservation of probabilities requires: $\sum _{j=1}^n Q_{ji}=1$ since each copy has to be either correct, $Q_{ii}$, or incorrect, $\sum _{j=1,j\ne i}^n Q_{ji}=1-Q_{ii}$. For the sake of simplicity, binary sequences will be considered here and we distinguish only between correct and incorrect base pairs.

A useful simplifying approximation is made by the uniform error rate model (Swetina 1982): The error per nucleotide and replication event, *p*, is assumed to be independent of the position of the nucleotide along the sequence and the nature of the nucleotide to be complemented. Then all elements of the mutation matrix can be expressed by a simple formula,

2 $$\begin{aligned} Q_{ji}\ =\ p^{d_{ij}}\,(1-p)^{l-d_{ij}}\ =\ p^l\varepsilon ^{d_{ij}}\ \ \mathrm {with}\ \ \varepsilon =\frac{p}{1-p}\,, \end{aligned}$$

with only three parameters: (i) the sequence length of the RNA molecules *l*, (ii) the mutation rate *p*, and (iii) the Hamming distance (Hamming 1950, 1986) between the two sequences interrelated by the mutation process, $d_{ij}=d_{\mathrm {H}}(X_i,X_j)$. Without changing important results for the purposes pursued here, the analysis of the model is substantially simplified by the assumption of constant chain lengths *l*, which is also consistent with the restriction to point mutations since point mutations do not change chain lengths by definition.

As an alternative to Eq. (1), mutation can be seen as a consequence of DNA change—damage and imperfect repair—during the whole life time of an organism, which is the idea in the Crow–Kimura mutation model (2009), pp 264–266]:

3 $$\begin{aligned} {{\mathsf{\mathbf A}}}+{{\mathsf{\mathbf X}}}_i+{{\mathsf{\mathbf E}}}\mathop {\rightarrow \rightarrow \rightarrow }\limits ^{k_i[{{\mathsf{\mathbf A}}}]}2{{\mathsf{\mathbf X}}}_i+{{\mathsf{\mathbf E}}}\ \ \mathrm {and}\ \ {{\mathsf{\mathbf X}}}_i\mathop {\rightarrow \rightarrow \rightarrow }\limits ^{\mu _{ji}}{{\mathsf{\mathbf X}}}_j\ . \end{aligned}$$

Interestingly, both models (1) and (3) although different with respect to the underlying physics give rise to identical mathematical problems (see e.g. Baake and Gabriel 1999 and Schuster 2016, pp 76–78).

### Deterministic and stochastic approaches

Reaction mechanisms are commonly analyzed by deterministic and stochastic approaches. The former translate the chemical reaction equations into kinetic differential equations, which can be directly solved by mathematics, studied by means of qualitative analysis or investigated by numerical integration. The theorems of existence and uniqueness of solutions of differential equations are applicable and a single integration provides the complete information for a given input set. Competitive selection with nonzero mutation leads to one unique asymptotically stable stationary state (Thompson 1974; Jones et al. 1976), whereas the long-time dynamics of cooperative systems is much richer and multiple stationary states, oscillations or deterministic chaos may be observed (Schuster 1978; Schnabl et al. 1991).

Stochastic analysis in general in based on searching for a stochastic process that fits the model to be studied as closely as possible. Chemical reaction kinetics prefers master equations although the repertoire of analytical solutions is very limited. It is not difficult to write down a multivariate master equation but the derivation of analytical solutions is successful only in exceptional cases, for example for networks of monomolecular reactions (Jahnke et al. 2007; Deuflhard et al. 2008. If no analytical solutions are available information on the stochastic system can be obtained by trajectory sampling. The theoretical background for trajectory harvesting has been laid down by Andrey Kolmogorov (1931), Willy Feller (1940), and Joe Doob (1942, 1945). With electronic computers now being generally available elaborate simulations of stochastic processes became possible. The more recent conception, analysis, and implementation of a simple but highly efficient algorithm by Daniel Gillespie (1976, 1977, 2007) provides a very useful tool for investigations of stochastic effects in chemical kinetics. Distributions of trajectories are characterized by expectation values and higher moments, commonly only by variances or standard deviations.

Equilibrium fluctuations in conventional chemical reaction kinetics follow an approximate $\sqrt{N}$-law and hence play almost no role in systems with particle numbers that are typical for chemical systems. In biology a different scenario is encountered. For example, every new variant originating from mutation has to start out from a single copy. The deterministic approach commonly uses continuous variables, which can only be an approximation to reality, since numbers of molecules or biological entities are integers by definition. Continuous concentrations can adopt arbitrarily small values whereas stochastic variables cannot pass low values beyond unity, because then molecular species go extinct, and deterministic solutions become unrealistic and differ strongly from the stochastic results. Large population size alone is not sufficient, each variable has to be sufficiently large at every instant to guarantee similarity between stochastic and deterministic solutions. Two more phenomena are relevant in the stochastic approach at low particle numbers and may lead to large standard deviations in the variables: (i) nucleation steps in reactions involving two or more molecules and (ii) multiple (quasi)stationary states^2^ of the stochastic system. The stochastic system may approach any stationary state and the distribution of the possible outcomes is determined by probabilities. Then the calculations of expectation values and higher distribution moments build averages over different final states and may give rise to enormous standard deviations.

For the purpose of illustration, we consider the equilibration of the flow reactor as an example of a stochastic system that can be fully analyzed by analytic calculations (Schuster 2016, pp 436–441). The two pseudoreactions,

4a $$\begin{aligned} *\ \ \ \mathop {\longrightarrow }\limits ^{a_0\,r}\ \ \ {{\mathsf{\mathbf A}}}\ \ \ \mathrm {and} \end{aligned}$$

4b $$\begin{aligned} {{\mathsf{\mathbf A}}}\ \ \ \mathop {\longrightarrow } \limits ^{r}\ \ \ \varnothing \ , \end{aligned}$$

are converted into the easy to solve kinetic differential equation

4c $$\begin{aligned} \frac{\mathrm {d}{a}}{\mathrm {dt}}\ =\ a_0\cdot r\,-\,a\cdot r\ =\ (a_0-a)\,r\ \ \mathrm {with}\ \ a(t)\ =\ n_0\,+\,\bigl (n_0-a_0\bigr )\,\mathrm{{e}}^{-r\,t} \end{aligned}$$

where $n_0=a(0)$. The master equation for the probability $P_n(t)=P\bigl (A(t)=n\bigr )$ with number density *A*(*t*) being the discrete pendant of concentration *a*(*t*),

4d $$\begin{aligned} \frac{\partial P_n}{\partial t}\ =\ r\bigl (a_0P_{n-1} +(n+1)P_{n+1}-(a_0+n)P_n\bigr )\,,\ n\in \mathbb {N}\,, \end{aligned}$$

has the analytical solution

4e $$\begin{aligned} P_n(t)\,=\,\sum _{k=0}^{\min \{n_0,n\}}\left( {\begin{array}{c}n_0\\ k\end{array}}\right) \,a_0^{n-k}\, \frac{\mathrm{{e}}^{-krt}(1-\mathrm{{e}}^{-rt})^{n_0+n-2k}}{(n-k)!}\,\mathrm{{e}}^{a_0(1-e^{-rt})}\ \end{aligned}$$

with $n,n_0,a_0\in \mathbb N$ for the *sharp* initial condition $P_n(0)=\delta _{n,n_0}$. In the limit $t\rightarrow \infty$ the distribution (4e) converges to a Poisson distribution

4f $$\begin{aligned} \lim _{t\rightarrow \infty }P_n(t)\ =\ \overline{P}_n\ =\ \frac{a_0^{\,n}}{n!}\,\exp (-a_0)\ . \end{aligned}$$

First and second moment yield expectation values and standard deviations

4g $$\begin{aligned} \mathrm {E}\bigl (A(t)\bigr)&=\ a_0\,+\,(n_0-a_0)\,\mathrm{{e}}^{-rt}\ \ \mathrm {and}\nonumber \\ \sigma \bigl (A(t)\bigr)&=\ \sqrt{(a_0+n_0\,\mathrm{{e}}^{-rt})(1-\mathrm{{e}}^{-rt})}\,. \end{aligned}$$

The standard deviation starts out from $\sigma (t=0)=0$ and approaches the equilibrium value $\overline{\sigma }=\sqrt{a_0}$ either from below for $a_0>n_0$ or from above for $a_0<n_0$ after having passed a maximum at

4h $$\begin{aligned} t_{\max }\,=\,\frac{1}{r}\ln \frac{2n_0}{n_0-a_0}\,. \end{aligned}$$

In general the maximum if it exists is very flat (see Fig. 3) as can be easily checked from the analytical expressions through inspection:

$$\begin{aligned} E\bigl (A(t_{\max })\bigr )\,=\,a_0+\frac{(n_0-a_0)^2}{2n_0}\ \ \mathrm {and}\ \ \sigma \bigl (A(t_{\max })\bigr )\,=\frac{n_0-a_0}{2\,\sqrt{n_0}}\,. \end{aligned}$$

The flow reactor is one of the few rather exceptional cases where the stochastic approach can be done completely by mathematical analysis.

**Fig. 2:**
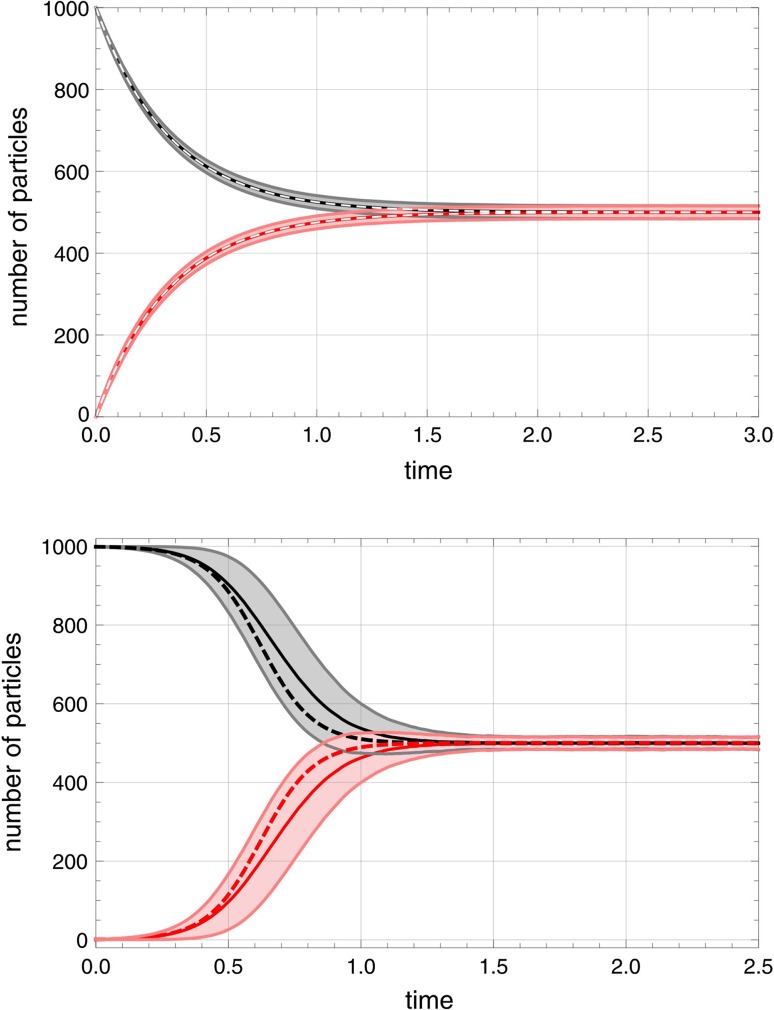
Comparison of fluctuations in the reversible zero- and first-order autocatalytic reaction ${{\mathsf{\mathbf A}}}+m{{\mathsf{\mathbf X}}}\rightleftharpoons (m+1){{\mathsf{\mathbf X}}}$ with $m=0,1$ bf in the batch reactor. The two plots show the expectation values $E\bigl (A(t)\bigr )$ (black) and $E\bigl (X(t)\bigr )$ (red) together with the one standard deviation band $E(t)\pm \sigma (t)$ (gray and pink) obtained by sampling of 10,000 trajectories that were calculated by Gillespie’s simulation method for the uncatalyzed ($m=0$; top plot) and the first-order autocatalytic reaction ($m=1$; bottom plot). Both reactions approach almost identical thermodynamic equilibrium states. Choice of parameters and initial conditions: $N=1000$; $h_{_{\rightarrow }}=h_{_{\leftarrow }}=1.5\,$[*t*$^{-1}$], A(0)=1000, X(0)=0 (top plot); $k_{_{\rightarrow }}=k_{_{\leftarrow }}=0.01\,$[$M^{-1}$ *t*$^{-1}$], A(0)=999, X(0)=1 (bottom). Solutions of the corresponding kinetic differential equations are shown as broken lines.

### Autocatalysis in the batch reactor

A batch reactor is an elaborate device that allows for performing chemical reactions under controlled conditions without inflow and outflow (Schmidt 2005). Here, the term batch reactor is used to indicate that reactions are carried out in a well-mixed closed system under constant temperature and pressure, and in the long run approach a thermodynamic equilibrium state.

In conventional chemistry autocatalysis is a rather rare phenomenon but in biology it represents the most important process since multiplication is just a special form of autocatalysis that in simplest form can be expressed by the reversible reaction

5 $$\begin{aligned} {{\mathsf{\mathbf A}}}\,+\,m\,{{\mathsf{\mathbf X}}}\ \ \ \mathop {\rightleftarrows }\limits _{g_\rightarrow }^{g_\leftarrow } (m+1)\,{{\mathsf{\mathbf X}}}\ . \end{aligned}$$

The forward reaction of (5) leads to molecular self-enhancement: The *resource* **A** is consumed in the synthesis of the replicator **X** and the process is catalyzed by the presence of further molecules **X**. Depending on the molecularity of the autocatalytic process as expressed by the value of *m* autocatalysis comes in different forms that, in essence, fall into two classes: (i) simple or first-order autocatalysis with $m=1$ and (ii) second- and higher-order autocatalysis with $m\ge 2$ [(Phillipson and Schuster 2009, pp 18–27)]. For consistency, we add here also the uncatalyzed reaction and call it *zeroth order autocatalytic* ($m=0$).^3^ All processes in closed systems converge to an equilibrium state and show mass conservation that implies a conservation relation $A(0)+X(0)=A(t)+X(t)=N$. The reversible autocatalytic reactions converge to the equilibrium state:

$$\begin{aligned} S_1\!\!: \overline{a}=N/(1+K),\ \overline{x}=N\cdot K/(1+K). \end{aligned}$$

Although the expressions for the equilibria are the same for the all reactions independently of the stoichiometric coefficient *m*, $K=g_{_{\rightarrow }}/g_{_{\leftarrow }}$, there are subtle differences in the probability distributions, which become important at small concentrations: The particle numbers are discrete quantities, change by integers only, and the stoichiometric factors are $X(t)\bigl (X(t)-1\bigr )\equiv \bigl (X(t)\bigr )_2$ or $X(t)\bigl (X(t)-1\bigr )\bigl (X(t)-2\bigr )\equiv \bigl (X(t)\bigr )_3$ rather than $X(t)^2$ and $X(t)^3$, respectively.^4^ The states with $X(t)=1$ for $m=1$, or the states $X(t)=1$ and $X(t)=2$ for $m=2$ cannot react because two or three molecules **X** are needed for the conversion of **X** into **A**. The inaccessibility of the state with $X(t)=0$ or the states with $X(t)=0$ and $X(t)=1$ in the first- or second-order autocatalytic reactions require different normalizations for the stationary probabilities of the three systems:

6a $$\begin{aligned} \overline{P}_n^{(0)}=\ \left( {\begin{array}{c}N\\ n\end{array}}\right) \ \frac{K^n}{(1+K)^N}\,;\ n\,\in \,[0,N]\ ,\end{aligned}$$

6b $$\begin{aligned} \overline{P}_n^{(1)}=\ \left( {\begin{array}{c}N\\ n\end{array}}\right) \ \frac{K^n}{(1+K)^N\,-\,K^N}\,;\ n\,\in \,[0,N-1]\ ,\end{aligned}$$

6c $$\begin{aligned} \overline{P}_n^{(2)}=\ \left( {\begin{array}{c}N\\ n\end{array}}\right) \ \frac{K^n}{(1+K)^N\,-\,K^N\,-\,N\,K^{N-1}-1}\,;\ n\,\in \,[1,N-2]\ , \end{aligned}$$

where as before the stochastic variable is $n=A$. Mass conservation provides $X=N-n$. As expected the truncation of $\overline{P}_n^{(0)}$ is important for small values of *N* only. For first-order autocatalysis with $N=10$, $k_{_{\rightarrow }}=k_{_{\leftarrow }}=1.0$ we obtain $\mathrm {E}(\overline{A})=4.9951$, for second-order autocatalysis $\mathrm {E}(\overline{A})=4.9605$ compared to $\mathrm {E}(\overline{A})=5$ for the uncatalyzed process.

**Fig. 3:**
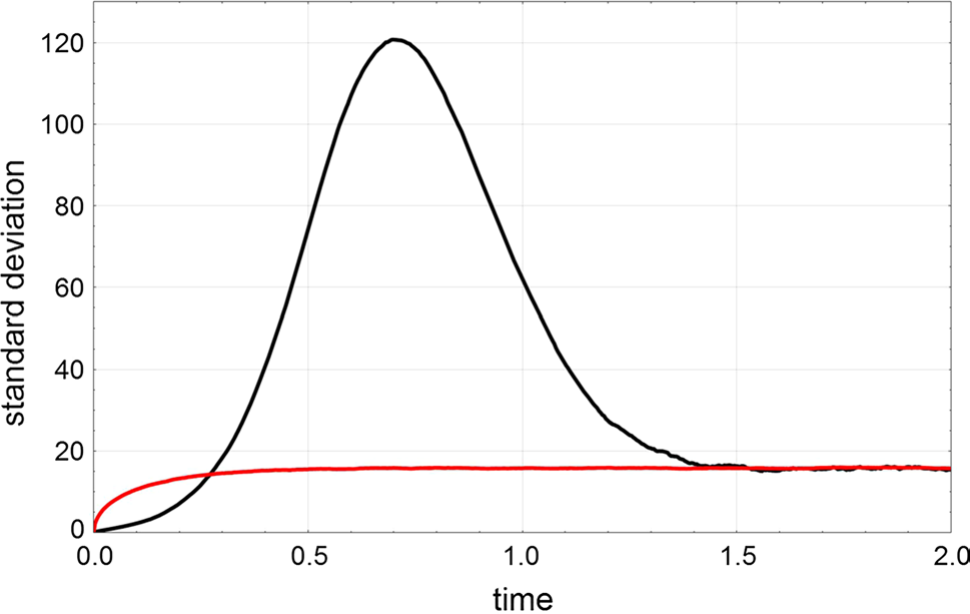
Comparison of the standard deviation in the reversible first-order autocatalytic reaction ${{\mathsf{\mathbf A}}}+{{\mathsf{\mathbf X}}}\rightleftharpoons 2{{\mathsf{\mathbf X}}}$ and the reversible uncatalyzed reaction ${{\mathsf{\mathbf A}}}\rightleftharpoons {{\mathsf{\mathbf X}}}$. The reactions are recorded for the closed system fulfilling $A(t)+X(t)=A_0+X_0=\mathrm {const}=N$ where $A(0)=A_0$ and $X(0)=X_0$ for both reactions. The figure presents the results of statistical evaluation of 10 000 trajectories obtained by computer simulations with Gillespie’s method (Gillespie 2007) for the autocatalytic reaction $\sigma _X^{(1)}(t)=\sigma ^{(1)}(t)$ (black) and for the uncatalyzed reaction $\sigma _X^{(0)}(t)=\sigma ^{(0)}(t)$ (red). Choice of parameters: $k_{_{\rightarrow }}=k_{_{\leftarrow }}=0.01\,$[$M^{-1}$ $t^{-1}$] and $h_{_{\rightarrow }}=h_{_{\leftarrow }}=1.5\,$[$t^{-1}$]; equilibrium parameters $K=k_{_{\rightarrow }}/k_{_{\leftarrow }}=h_{_{\rightarrow }}/h_{_{\leftarrow }}=1$; initial conditions: $N=1000$, $X_0=1$, $A_0=999$ and $N=1000$, $X_0=0$, $A_0=1000$ yielding the numerical equilibrium values $\overline{A}=\overline{X}=500$. The equilibrium value of the standard deviation is practically the same for both reactions: $\lim _{t\rightarrow \infty }\sigma _X^{(1)}(t)\approx \lim _{t\rightarrow \infty }\sigma _X^{(0)}(t)\approx 15.8114$

In principle, there are three major sources of randomness in autocatalytic reactions: (i) thermal fluctuations, (ii) delayed onset of autocatalytic reactions and (iii) multiple stationary states. (ad i) In the transients towards equilibrium the thermal fluctuation bands are essentially the same in autocatalytic and conventional reactions as can be seen best from the comparison of standard deviations at equilibrium (Fig. 2). The differences between conventional and autocatalytic equilibrium densities can be recognized numerically for very small particle numbers only (). (ad ii) The reaction rate for an autocatalytic reaction of order *m* is $v(0)=k\,A(0)\,\bigl (X(0)\bigr )_m\,-\,l\,\bigl (X(0)\bigr )_{m+1}$ (with $m\ge 1$). At small *t* the factor *A*(*t*) is large and the factor(s) containing *X*(*t*) are small and only the first term in *v*(*t*) matters in the early phase of the reaction. Thus **X** is produced and *X*(*t*) increases, which in turn leads to an increase *v*(*t*) yielding the typical scenario of self-enhancement. Self-enhancement in chemical reactions is tantamount to an increase of the reaction rate with concentration in the early phase and together with the late saturation phase gives rise to *“s”*-shaped or *sigmoid* curves whereas the uncatalyzed reaction ($m=0$) follows a simple exponential decay (Fig. 2). Higher values of *m* lead to steeper curves, which approach a step function with increasing *m*. The maximum standard deviation in the approach towards equilibrium $\sigma _{\max }=\max \{\sigma (t)\}$ is a measure of the random scatter in the delay in the onset of the autocatalytic reaction (Table 1). (ad iii) Multiple final states give rise to an additional stochastic component often called anomalous fluctuations (de Pasquale et al. 1980) since "Autocatalysis in the ow reactor").

**Table 1:** Fluctuations in the two autocatalytic reactions **A**+ **X** $\rightleftharpoons$2 **X** **and** **A**+2 **X** $\rightleftharpoons$3 **X** $^a$

Autocatalysis order	Reaction	Initial conditions				Limit
		$X_0=1$	$X_0=2$	$X_0=5$	$X_0=10$	$t\rightarrow \infty$
Zero	**A** $\rightleftharpoons$ **X**	–	–	–	–	15.8$^b$
First	**A**+ **X** $\rightleftharpoons$2 **X**	123.2	89.8	59.6	44.4	15.8$^b$
Second	**A**+2 **X** $\rightleftharpoons$3 **X**	–	245.1	226.7	189.4	15.8$^b$

The table presents maximal standard deviations $\sigma _{\max }$ computed from 1 000 trajectories of the two autocatalytic reactions for different initial conditions $X_0=X(0)$. For the autocatalytic reactions the standard deviation $\sigma (t)$ passes through the maximum $\sigma _{\max }$ listed here whereas for the uncatalyzed process it increases monotonously from $\sigma (0)=0$ to the equilibrium value (see Fig. 3)^a^

The equilibrium fluctuations calculated from equations () are practically the same for all three reactions. Choice of parameters, $N=1000$, **A** $\rightleftharpoons$ **X**: $h_{_{\rightarrow }}=h_{_{\leftarrow }}=1.5\,$[t$^{-1}$]; **A**+ **X** $\rightleftharpoons$2 **X**: $k_{_{\rightarrow }}=k_{_{\leftarrow }}=0.01\,$[M$^{-1}$ t$^{-1}$]; **A**+2 **X** $\rightleftharpoons$3 **X**: $l_{_{\rightarrow }}=l_{_{\leftarrow }}=0.00001\,$[M$^{-2}$ t$^{-1}$]

^a^Depending on rate parameters and initial conditions the trajectories may pass a flat maximum before they decrease to the equilibrium value (see Eq. (4h) and Schuster 2016, pp. 445–449)

^b^ The accurate value obtained from the stationary master equation is $\sigma =15.8114$

In Fig. 2 transients for the two processes ${{\mathsf{\mathbf A}}}+m{{\mathsf{\mathbf X}}}\rightleftharpoons (m+1){{\mathsf{\mathbf X}}}$ with $m=0$ and 1 are compared by means of expectation values and fluctuation bands. As initial conditions we apply an empty reactor, $A(0)=0$, and the smallest possible values for **X**: $X(0)=0$ and $X(0)=1$ for the uncatalyzed and the autocatalytic reaction, respectively. A total population size of $N=1000$ was chosen so that the one-standard-deviation fluctuation band of order $\sqrt{N}$ appears small and the deterministic solutions coincide with the expectation values in the reference process **A** $\rightleftharpoons$ **X** ($m=0$). The transient of the autocatalytic process ($m=1$) is different: It shows substantial broadening of the fluctuation band (Table 1) because of delayed onset of the reaction before it narrows down to the equilibrium value. In the intermediate range the expectation values differ remarkably from the deterministic solution curves. As expected an increase in $X_0$ leads to a decrease in the width of the fluctuation band. Second order autocatalysis ($m=2$) differs from first-order autocatalysis mainly by the size of the characteristic effects: Both autocatalytic reactions show broadening of the fluctuation bands but the band width in the second-order case is about four times as wide. In particular, the scatter in the waiting times until the first reaction events occurs is much larger because we are dealing with two small factors, $(X-1)\cdot (X-2)$, in the expression for *v*(0).

The standard deviation in the course of the reactions, $\sigma (t)$, is shown in Fig. 3. Because of sharp initial conditions the fluctuation band starts out from zero—$\sigma (0)=0$, increases, becomes broad in the intermediate range and settles down at the equilibrium value (6). Substantial deviations between the deterministic solution and the stochastic expectation value, *a*(*t*) and the $E\bigl (A(t)\bigr )$ or *x*(*t*) and $E\bigl (X(t)\bigr )$, respectively, are observed in the intermediate range. Accordingly, the standard deviation goes through a pronounced maximum that is qualitatively different from the shallow maximum observed with the standard deviation of conventional chemical processes $\bigl ($see Eq. (4h)$\bigr )$.

The irreversible processes, ${{\mathsf{\mathbf A}}}+m{{\mathsf{\mathbf X}}}\rightarrow (m+1){{\mathsf{\mathbf X}}}$ obtained from Eq. (5) by putting $g_{_{\leftarrow }}\!\!=0$, are illustrative because they are closer to biology where replication or reproduction occur always under the conditions of irreversibility. The shape of the solution curves compared to those shown in Fig. 2 shows similarities except a twice as wide range for the values of the stochastic variables, $A(0)+X(0)\ge A(t)\ge 0$ and $1\le X(t)\le A(0)+X(0)$ and expectation values and standard deviations approach zero at sufficiently long times. Again, we observe a sigmoid shape of the solution curves, for small initial values ($X(0)<5$) the standard deviation $\sigma (t)$ becomes large in the intermediate range (Fig. 3), and the deterministic curve deviates substantially from the stochastic expectation values.

**Fig. 4:**
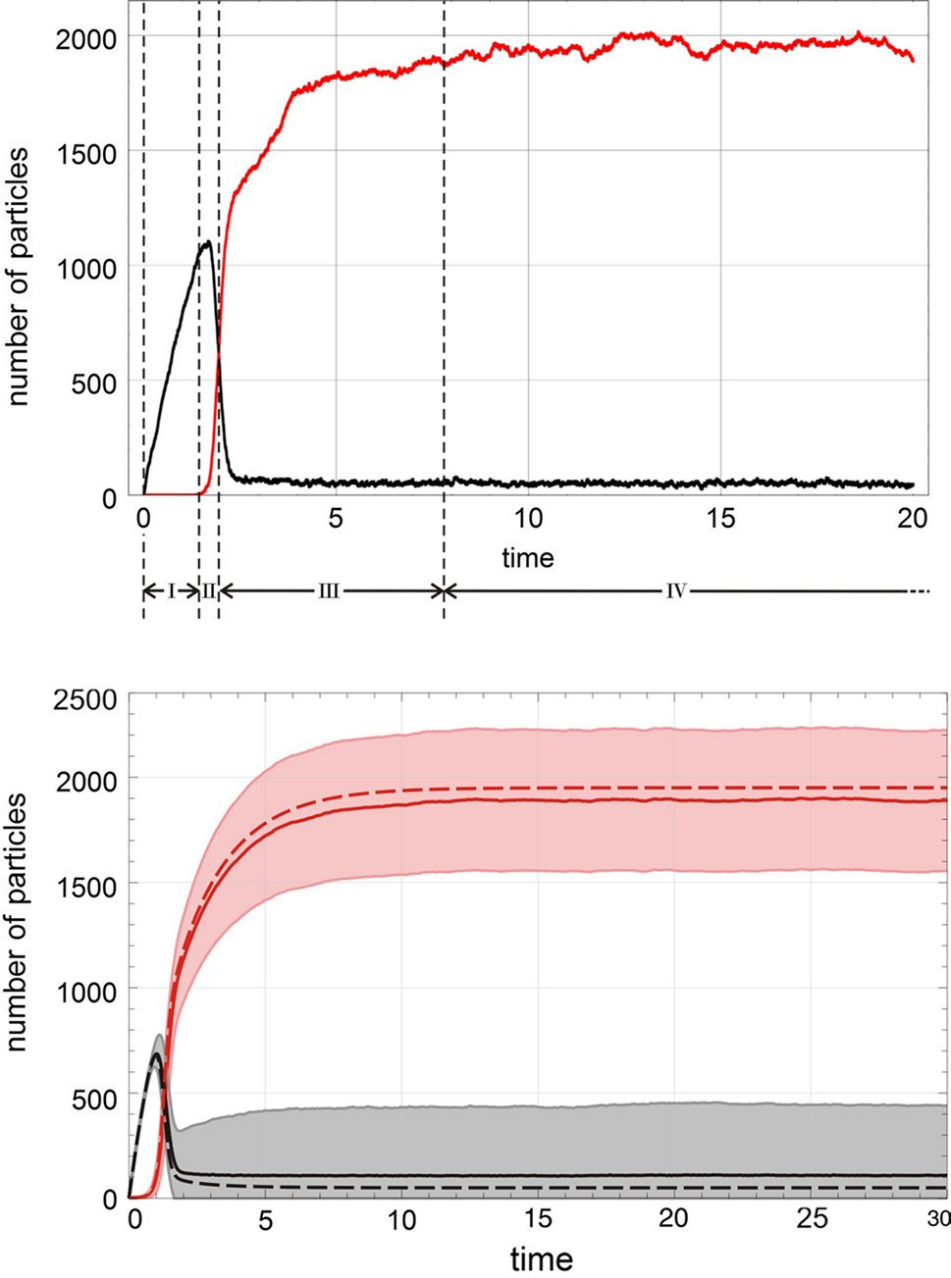
The first-order autocatalytic reaction. ${{\mathsf{\mathbf A}}}+{{\mathsf{\mathbf X}}}\rightleftharpoons 2{{\mathsf{\mathbf X}}}$ in the flow reactor. The two plots show a single trajectory (top plot) and statistics consisting of expectation value within the one-standard deviation band, $E\bigl (A(t)\bigr )\pm \sigma \bigl (A(t)\bigr )$ and $E\bigl (X(t)\bigr )\pm \sigma \bigl (X(t)\bigr )$ taken form sample of 100 trajectories (bottom plot). The four phases of the approach to the long-time solution are indicated (top plot; see text). Choice of parameters and initial conditions: $N=2000$; $k=0.01\,$[M$^{-1}$ t$^{-1}$], $r=0.5\,$[V t$^{-1}$], $A(0)=0$, $X(0)=1$ (top) and $X(0)=3$ (bottom). Pseudorandom number generator: *ExtendedCA, Mathematica*, seeds $s=491$. The sample size of the bottom plot was 100 trajectories, 3 led to $S_0$ (extinction) and 97 reached the pseudo-stationary state $S_1$. Solutions of the corresponding kinetic differential equations are shown as dashed lines

### Autocatalysis in the flow reactor

Implementation of autocatalytic reactions in the flow reactor provides additional insights into the different forms of randomness. In particular we are interested in multiple stationary states as a source of stochasticity (item iii). The reaction equations for first order autocatalysis are:

7a $$\begin{aligned} *\ \ \ \mathop {\longrightarrow }\limits ^{a_0\,r}\ \ \ {{\mathsf{\mathbf A}}}, \end{aligned}$$

7b $$\begin{aligned} {{\mathsf{\mathbf A}}}\,+\,{{\mathsf{\mathbf X}}}\ \ \ \mathop {\longrightarrow }\limits ^{k}\ \ \ 2\,{{\mathsf{\mathbf X}}}, \end{aligned}$$

7c $$\begin{aligned} {{\mathsf{\mathbf A}}}\ \ \ \mathop {\longrightarrow }\limits ^{r}\ \ \ \varnothing,\ \ \mathrm {and} \end{aligned}$$

7d $$\begin{aligned} {{\mathsf{\mathbf X}}}\ \ \ \mathop {\longrightarrow }\limits ^{r}\ \ \ \varnothing, \end{aligned}$$

with the kinetic differential equations

7e $$\begin{aligned} \frac{\mathrm {d}{a}}{\mathrm {dt}}\ =\ -\,k\,a\,x\,+\,(a_0-a)\,r\ \ \mathrm {and}\ \ \frac{\mathrm {d}{x}}{\mathrm {dt}}\ =\ x\,(k\,a-r). \end{aligned}$$

The simple first-order autocatalytic process in the flow reactor sustains two long time states: (i) The *state of extinction* $S_0$ with $\lim _{t\rightarrow \infty } a(t)=\overline{a}=a_0$ and $\lim _{t\rightarrow \infty } x(t)=\overline{x}=0$, and (ii) the quasistationary state $S_1$ with $\lim _{t\rightarrow \infty } a(t)=\overline{a}=r/k$ and $\lim _{t\rightarrow \infty } x(t)=\overline{x}=a_0-r/k$. Stability of $S_1$ requires that the condition $r<a_0\,k$ is fulfilled.

Starting from an empty reactor containing no **A** and the autocatalyst only in seeding quantities, $A(0)=0$ and $X(0)=1,2,3,\ldots$ , the trajectory shown in the upper part of Fig. 4 allows for the distinction of several phases: (I) the flow reactor is filled with the resource **A** in phase I, (II) in the random phase II the decision is made to which state the trajectory will converge, (III) the trajectory approaches the long-time state, and (IV) the trajectory fluctuates around the state (see Figs. 4 and 7). First-order autocatalysis sustains the two long-time solutions $S_0=(\overline{a}^{(0)},0)$ and $S_1=(\overline{a}^{(1)},\overline{x}^{(1)})$, stochastic trajectories approach either of the two states, and parameters and initial conditions determine the probabilities to end up here or there. For sufficiently large population sizes, the long-time expectation values of the stochastic variables can be well approximated by linear combination of the deterministic values:

$$\begin{aligned} \mathrm {E}\bigl (\overline{A}\bigr )=\xi _0\overline{a}^{(0)}+\xi _1\overline{a}^{(1)} {\mathrm{and}} \mathrm {E}\bigl (\overline{X}\bigr )=\xi _0\overline{x}^{(0)}+\xi _1\overline{x}^{(1)} \end{aligned}$$

with $\xi _0=N_0/(N_0+N_1)$ and $\xi _1=N_1/(N_0+N_1)$ where $N_0$ and $N_1$ are the counted numbers of trajectories ending up in $S_0$ or $S_1$ from a sufficiently large sample. Although only 3/100 trajectories go to extinction in the example shown in the lower plot of Fig. 4 the influence on the expectation values $E\bigl (A(t)\bigr )$ and $E\bigl (X(t)\bigr )$ and the standard deviations $\sigma \bigl (A(t)\bigr )$ and $\sigma \bigl (X(t)\bigr )$ is remarkable. This random component of processes has been intensively studied in the nineteen eighties by Paolo Tombesi, Francesco de Pasquale, Piero Tartaglia and the notion anomalous fluctuation caused by a chemical instability was coined for this kind of stochasticity (de Pasquale et al. 1980, 1982, 1982). It is advantageous to collect trajectories separately for the different final states, because then the anomalous fluctuations disappear. For example, the standard deviation of **A** at $t=30$ is reduced from $\sigma \bigl (A(30)\bigr )=335.7$ to $\sigma \bigl (A(30)\bigr )=7.12$ if one changes from a sample of hundred trajectories with three extinction events (Fig. 4, Pseudorandom number generator: *ExtendedCA, Mathematica*, seeds $s=491$) to one without extinction ($s=919$).

The major difference between the two classes of autocatalytic reactions lies in the repertoire of possible dynamic behaviors. First-order autocatalysis gives rise to exponential growth in unconstrained systems and to selection and optimization of mean fitness in multispecies cases with finite resources (see ''Competition, mutation and quasispecies"). Accordingly first-order autocatalysis leads to a Darwinian scenario of selection of the fittest. In contrast to first-order autocatalytic reaction networks, the dynamics in second-order systems is very rich and includes multiple stationary states, oscillations and deterministic chaos. The second-order autocatalytic elementary step, **A**+2 **X** $\rightleftharpoons$3 **X**, represents a kind of generally used prototype for theoretical models, for example the Brusselator (Nicolis and Prigogine 1977). It provides a simple enough reaction step for studies by means of rigorous mathematics. Qualitative analysis of Brusselator dynamics is straightforward and numerical integration causes no problem provided the integration software can handle stiff differential equations. In reality, however, single-step autocatalytic reactions are extremely rare, instead we are commonly dealing with multistep-reaction networks (Noyes et al. 1972) (see also the review by Francesc Sagués and Irving Epstein (2003).

In biology, in particular in the theory of evolution, the process **A**+2 **X** $\rightarrow$3 **X** plays a special role since in the simple form of hypercycles it is the basis for suppression of competitive selection without destroying template-induced reproduction. It provides one fundamental mechanism for major transitions (Maynard Smith and Szathmáry 1995; Schuster 1996) and will be discussed extensively in "Competition and cooperation". The enormous flexibility of second-order autocatalysis follows, for example, from Fisher’s selection equation and the proof for the optimization of mean fitness in sexual reproduction under idealized condition. In a caricature model we may explain how the above mentioned reaction step could be related to sexual reproduction: 2 **X** on the l.h.s. are (at least stoichiometrically) related to the parents and 3 **X** on the r.h.s. model parents and one offspring. Apart from being illustrative toys autocatalytic processes set the stage for modeling evolution in the sense that we shall reencounter all special phenomena of autocatalysis in the more elaborate model for evolution to be presented and discussed in the next "A minimal mathematical model for the evolution of molecules".

**Fig. 5:**
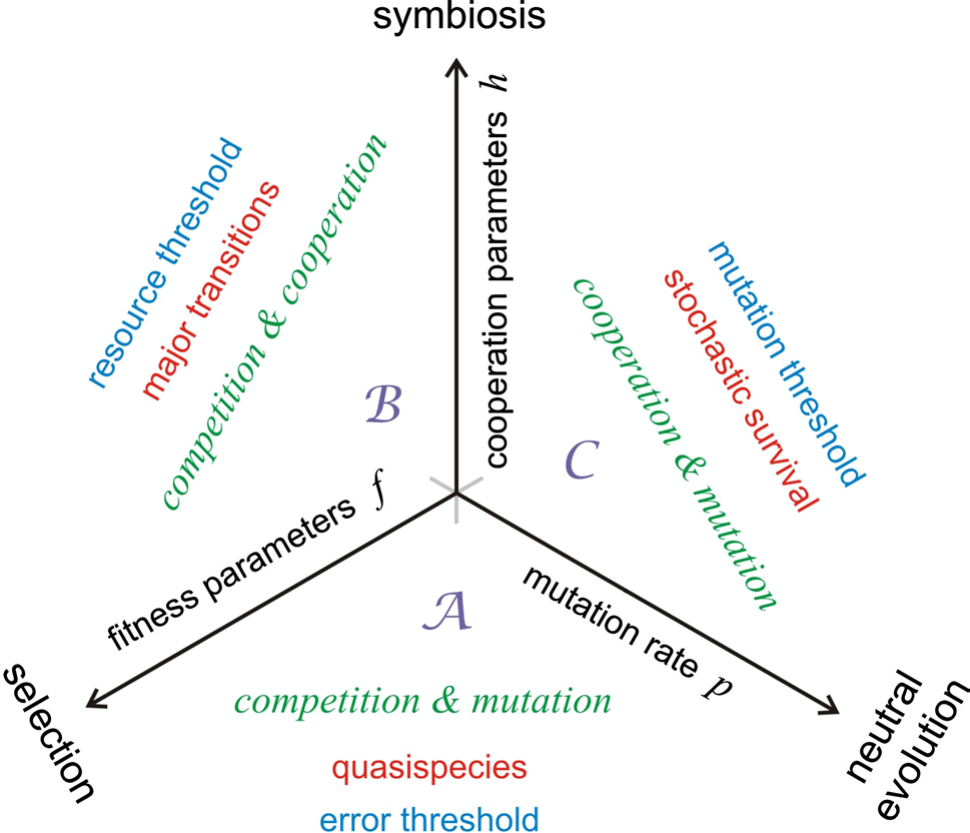
A minimal model for modeling evolution. Evolution is considered as an interplay of three processes: (i) competition through reproduction, (ii) cooperation through symbiosis, and (iii) mutation through error-prone replication. In parameter space, the intensity parameters of all three processes, (i) fitness parameters *f* corresponding to reaction rates for competition, (ii) reaction rates *h* for catalyzed reproduction, and (iii) an error rate parameter *p* for mutation are plotted on the axes of a Cartesian coordinate system. On the three two-dimensional faces of the coordinate system we are dealing with the three fundamental evolutionary processes: (${\mathcal A}$) competitive reproduction and mutation are the basis of Darwinian optimization through natural selection and give rise to the formation of quasispecies and eventually to the occurrence of error thresholds (Eigen 1971; Eigen and Schuster 1977; ${\mathcal B}$) the interplay of competition and cooperation allows for the description of major transitions, which are seen as the consequences of crossing resource thresholds (Maynard Smith and Szathmáry 1995; Szathmáry and Maynard Smith 1995; Schuster 1996, 2016); (${\mathcal C}$) the combination of cooperation and mutation enables reintroduction of extinguished species provided the error rate is sufficiently large such that a mutation threshold for stochastic survival can be recognized (Schuster 20160

## A minimal mathematical model for the evolution of molecules

The minimal model is dealing with the time dependence of the distribution of genotypes in populations ${\Uppi }(t)$ and hence it is rooted in chemical kinetics of (i) competitive reproduction, (ii) symbiontic cooperation, and (iii) genetic variation. The quantification of these three properties yields three parameters or sets of parameters, which can be plotted on the three axes of a Cartesian coordinate system (see Fig. 5 and the previous publications (Schuster 2016a, b, d, 2017b). We consider the simplest case here, where the three quantities are a fitness parameter *f*, a cooperation parameter *h*, and an mutation rate parameter *p*. For an implementation of the model in the flow reactor we need two additional external parameters measuring the accessible resources expressed, for example, as number density or concentration of a (hypothetical) compound **A**, $A_0$ or $a_0$, respectively, and the mean residence time $\tau _{\mathrm {R}}=V/r$ of the reaction mixture in the reactor where *V* is the volume of the reactor and *r* is the (volumetric) flow rate. The parameter $\tau _{\mathrm {R}}$ defines the time resolution of the reactor since slow reactions, which do not progress appreciably during the time interval $\tau _{\mathrm {R}}$ cannot be studied.

In the next step, the model is implemented by means of a suitable and simple reaction mechanism. Based on "Idealized environments" and "Basic processes" we consider the following set of $2n^2+n+2$ reactions in the flow reactor:

8a $$\begin{aligned} *\ \ \ \mathop {\longrightarrow }\limits ^{a_0\,r}\ \ \ {{\mathsf{\mathbf A}}}\,; \end{aligned}$$

8b $$\begin{aligned} {{\mathsf{\mathbf A}}}\,+\,{{\mathsf{\mathbf X}}}_i\ \ \ \mathop {\longrightarrow }\limits ^{k_i\,Q_{ji}}\ \ \ {{\mathsf{\mathbf X}}}_i\,+\,{{\mathsf{\mathbf X}}}_j\, ,\ i,j=1,\ldots ,n\,; \end{aligned}$$

8c $$\begin{aligned}{{\mathsf{\mathbf A}}}\,+\,{{\mathsf{\mathbf X}}}_i\,+\,{{\mathsf{\mathbf X}}}_{i+1}\ \ \ \mathop {\longrightarrow }\limits ^{l_i\,Q_{ji}}\ \ \ {{\mathsf{\mathbf X}}}_i\,+\,{{\mathsf{\mathbf X}}}_j+\,{{\mathsf{\mathbf X}}}_{i+1}\ , i,j=1,\ldots ,n\,;\ i\!\!\!\!\mod n\, ; \end{aligned}$$

8d $$\begin{aligned} \nonumber \\ {{\mathsf{\mathbf A}}}\ \ \ \mathop {\longrightarrow }\limits ^{r}\ \ \ \varnothing \ ;\ \ \mathrm {and} \end{aligned}$$

8e $$\begin{aligned} {{\mathsf{\mathbf X}}}_i\ \ \ \mathop {\longrightarrow }\limits ^{r}\ \ \ \varnothing \,,\ i=1,\ldots ,n . \end{aligned}$$

The process (8a) supplies the material required for reproduction. A solution with **A** at concentration $a_0$ flows into a continuously stirred tank reactor (CSTR) with a flow rate parameter *r* (Schmidt 2005, p. 87ff). The reactor operates at constant volume and this implies that the volume of solution flowing into the reactor per time unit [*t*] is compensated exactly by an outflow, which is described by the Eqs. (8d) and (8e) and concerns all $(n+1)$ molecular species, **A** and ${{\mathsf{\mathbf X}}}_i\,,\ i=1,\dots ,n$. Inflow and outflow are often characterized as *pseudoreactions* because they are no chemical reactions in the strict sense, which are converting reactants are into products. The two classes of reactions producing progeny, template induced replication (8b) and catalyzed template induced replication (8c), represent the core of the evolution model. In agreement with the conditions in biology, both reproduction steps are implemented irreversibly in the direction of polynucleotide synthesis. A basic assumption for both reproduction steps is that correct reproduction and mutation are parallel chemical reaction channels ("Basic processes"). In other words, there is no mutation under conditions that do not sustain reproduction.

As an alternative to the Eigen model (1) mutation can be seen, for example, as the result of DNA damage and imperfect damage repair during the whole life span of an organism, which is the idea underlying the Crow–Kimura mutation model (Crow and Kimura 2009, pp 264–266). Then reproduction and mutation are completely independent processes,

$$\begin{aligned} {{\mathsf{\mathbf A}}}\,+\,{{\mathsf{\mathbf X}}}_i\ \mathop {\longrightarrow }\limits ^{k_i}\ \ 2\,{{\mathsf{\mathbf X}}}_i\quad \mathrm {and}\quad {{\mathsf{\mathbf X}}}_i\ \ \mathop {\longrightarrow }\limits ^{\upmu _{ji}}\ \ {{\mathsf{\mathbf X}}}_j\ , \qquad \qquad \qquad \mathrm{(8b')} \end{aligned}$$

and in the kinetic differential equations they appear as additive terms. Interestingly, the Eigen and the Crow–Kimura model although being fundamentally different with respect to the assumptions about the nature of the mutation process give rise to the same mathematical problems [see, e.g., (Schuster 2016d, pp.76-78)]. The mutation matrix $\mathrm {Q}$ corresponds formally to the mutation matrix ${\varvec{\upmu }}$ but there are non-negligible differences $\mathrm {Q}$ covers correct and error-prone replication but the process ${{\mathsf{\mathbf X}}}_i\rightarrow 2{{\mathsf{\mathbf X}}}_i$ is handled separately in the Crow–Kimura model and hence all diagonal terms are zero $\upmu _{ii}=0\,\forall \,i=1,\ldots ,n$.

The equations that will be applied in the analysis of the dynamics of the model (8) implement three processes along the coordinate axes: (i) Darwinian selection of the fittest on the competition axis, (ii) hypercycle dynamics on the cooperation axis, and (iii) neutral evolution on the mutation axis. The kinetic differential equations of the model mechanism (8) are of the form:

9a $$\begin{aligned} \frac{\mathrm {d}{a}}{\mathrm {dt}}\ = -a\,\sum _{i=1}^n\bigl (k_i\,+\,l_i x_{i+1}\bigr )\,x_i\ +\ r\,(a_0-a)\,,\ i\!\!\!\!\mod n\ \mathrm {and}\end{aligned}$$

9b $$\begin{aligned} \frac{\mathrm {d}{x_i}}{\mathrm {dt}}\ = a\left( \sum _{j=1}^n Q_{ij}\bigl (k_j\,+\,l_j x_{j+1}\bigr )\,x_j\right) \,-\,x_i\,r\ ,\\ \ \ i=1,2,\ldots ,n;\ j\!\!\!\!\mod n\,.\nonumber \end{aligned}$$

In (9a) we made use of the conservation relation $\sum _{i=1}^n Q_{ij}=1$. No analytical solutions are available for  (9) in general but numerical integration is straightforward as long as *n* is not too large. In absence of cooperation terms, $l_i=0\,\forall \,i=1,\ldots ,n$, Eq. (9) can be transformed into an eigenvalue problem of a symmetric matrix, which is readily diagonalized provided *n* is not very large ($n<10^6$; "Competition, mutation and quasispecies").^5^ In mutation-free systems, $p=0$ ("Competition and cooperation"), qualitative analysis and determination of stationary states are straightforward, and the dynamics of the complete system can be derived by extrapolation from the error-free results to finite mutations rates. The cooperation system with mutation, (9) with $k_i=0\,\forall \,i=1,\ldots ,n$ is used here to study the relevance of mutation in symbiontic systems. It also serves as an example for the study of unconventional consequences of replication with frequent errors in the strong mutation scenario ("Cooperation and mutation").

### Processes on individual coordinate axes

The processes along the individual coordinate axes are considered in order to verify the initial statement on the three basic processes. For this goal it is easiest to set certain parameters zero: (I) no natural selection implies $k_1=k_2=\ldots =k_n=k\,(=0)$, (II) no cooperative coupling requires $l_1=l_2=\ldots =l_n=l=0$, and (III) no mutation leads to $\mathrm {Q}={\mathbb I}$ where ${\mathbb I}$ is the unit matrix.

A process taking place on the selection axis is given by (II) and (III) being true leads to the ODE

10a $$\begin{aligned} \frac{\mathrm {d}{a}}{\mathrm {dt}}\ =-a\,\sum _{i=1}^n k_i\,x_i\ +\ r\,(a_0-a)\ \ \mathrm {and}\end{aligned}$$

10b $$\begin{aligned} \frac{\mathrm {d}{x_i}}{\mathrm {dt}}\ =\ \ (k_i\,a\,-\,r)\,x_i\,;\ i=1,2,\ldots ,n\,. \end{aligned}$$

Competition between the reproducing elements ${{\mathsf{\mathbf X}}}_i$ leads to *survival of the fittest subspecies* ${{\mathsf{\mathbf X}}}_m$, which is defined by $k_m\,a=f_m=\max \{f_i;i=1,\ldots ,n\}$. For constant $[{{\mathsf{\mathbf A}}}]=a_0=\mathrm {const}$, the mean fitness of the population, $\phi (t)=\sum _{i=1}^n f_i x_i(t)\!\bigm /\!c(t)$ with $c(t)=\sum _{i=1}^n x_i(t)$, is a non-decreasing function of time: ${\mathrm d}\phi /{\,\mathrm {dt}}=\mathrm {var}\{f\}\ge 0$. This result is the formal analogue of Fisher’s fundamental theorem for asexual reproduction.

On the cooperation axis, the conditions (I) and (III) are fulfilled and we obtain the equations of hypercycle dynamics

11a $$\begin{aligned} \frac{\mathrm {d}{a}}{\mathrm {dt}}\ =-a\,\sum _{i=1}^n\,l_i\,x_i\,x_{i+1}\ +\ r\,(a_0-a)\ \ \mathrm {and}\end{aligned}$$

11b $$\begin{aligned} \frac{\mathrm {d}{x_i}}{\mathrm {dt}}\ = \ \ \ (l_i\,x_{i+1}\,a\,-\,r)\,x_i\,,\ \ i=1,2,\ldots ,n,\ i\!\!\!\mod n\,, \end{aligned}$$

which were studied in great detail in a number of previous papers (Eigen and Schuster 1978; Schuster 1978; Schuster et al. 1979, 1980; Hofbauer et al. 1991).

The third case—with (I) and (II) being true—yields a degenerate deterministic solution: All distributions of subspecies with fixed population size $\sum _{i=1}^n x_i=c$ yield the same solutions and therefore constitute an $(n-1)$-dimensional invariant manifold fulfilling

12a $$\begin{aligned} \frac{\mathrm {d}{a}}{\mathrm {dt}}\ = -a\,k\,c\ +\ r\,(a_0-a)\ \ \mathrm {and}\end{aligned}$$

12b $$\begin{aligned} \frac{\mathrm {d}{c}}{\mathrm {dt}} &= a\,k\,c\ -\ c\,r\ =\ (a\,k\,-\,r)\,c\ \ \text {and}\\ \frac{\mathrm {d}{\gamma }}{\mathrm {dt}}\ &=\ (a_0-\gamma )\,r\ \ \mathrm {with}\ \ \gamma \,=\,a+c\,\end{aligned}$$

In the absence of selection and cooperation neutral evolution in the sense of Motoo Kimura (1955, 1983) is observed on the mutation axis. For an adequate description of the process a stochastic treatment is required. Random selection of a single arbitrarily chosen subspecies is observed in the no-mutation limit, $\lim p\rightarrow 0$. For non-vanishing mutation rates once selected subspecies may be replaced by other subspecies and the mean time of replacement of one randomly selected subspecies is $\overline{T}_{\mathrm {rep}}=\mu ^{-1}$ where $\mu$ is the mutation rate per generation (Kimura 1955, 1983). Translated into our model, we find for single point mutations $\mu \approx p$.

### Master equation and simulation

Reaction  (8) can be cast into chemical master equations.The particle numbers of the molecular species, $[{{\mathsf{\mathbf A}}}]=A(t)$ and $[{{\mathsf{\mathbf X}}}_i]=X_i(t)$ with $i=1,\ldots ,n$, are integers and in the absence of flows they fulfil the conservation relations, $A(t)+\sum _{i=1}^n X_i(t)=C$. The variables of the master equation are the probabilities

13 $$\begin{aligned} P_m(t)\,=\,\mathrm {Prob}\,\Bigl (A(t)=m\Bigr )\ \ \mathrm {and}\ \ P_{s_i}(t)\,=\,\mathrm {Prob}\,\Bigl (X_i(t)=s_i\Bigr )\,;\ i=1,\ldots ,n\,, \end{aligned}$$

and the indices are subsumed in an index vector, ${\mathbf m}=(m,s_1,\ldots ,s_n)$, which characterizes the state $S_{{\mathbf m}}$ of the system. The chemical master equation is based on the assumption that chemical processes occur one at a time, all jumps involve single steps and the particle numbers change by $\pm 1$ unless the elementary steps involve more than a single molecule per class. The jumps $S_{{\mathbf m}^{\,\prime }}\rightarrow S_{{\mathbf m}}$ or $S_{{\mathbf m}}\rightarrow S_{{\mathbf m}^{\,\prime }}$ are denoted by the shorthand notation

$$\begin{aligned} {\mathbf m}^{\,\prime }\,&=\,(m\pm 1,s_1,\ldots ,s_n)\equiv ({\mathbf m};\,m\pm 1)\ \ \mathrm {or}\\ {\mathbf m}^{\,\prime }\,&=\,(m,s_1,\ldots ,s_k\pm 1,\ldots ,s_n)\equiv ({\mathbf m};\,s_k\pm 1). \end{aligned}$$

Then the master equation of mechanism (8) takes on the form

14 $$\begin{aligned}&\frac{\mathrm {d}{P_{{\mathbf m}}}}{\mathrm {dt}}\ =\ \ a_0r\Bigl (P_{({\mathbf m};m-1)}-P_{{\mathbf m}}\Bigr )\,+\,r\Bigl ((m+1)P_{({\mathbf m};m+1)}-mP_{{\mathbf m}}\Bigr )\nonumber \\&\qquad \quad \quad \ +\,r\sum _{i=1}^n\Bigl ((s_i+1)P_{({\mathbf m};s_i+1)}-s_i P_{{\mathbf m}}\Bigr )\nonumber \\&+\,\sum _{i=1}^n Q_{ii}(k_i+l_i\,s_{i+1})\Bigl ((m+1)(s_i-1)P_{({\mathbf m};m+1,s_i-1)}-m\,s_i P_{{\mathbf m}}\Bigr )\nonumber \\&+\,\sum _{i=1}^n\sum _{j=1,j\ne i}^n Q_{ij}(k_j+l_j\,s_{j+1})s_j\Bigl ((m+1)P_{({\mathbf m};m+1,s_i-1)}-mP_{{\mathbf m}}\Bigr )\ . \end{aligned}$$

Each reaction step involving $S_{{\mathbf m}}$ changes the probability to be in state $S_{{\mathbf m}}$, $P_{{\mathbf m}}$, in two ways: It increases the probability through reactions or pseudoreactions $S_{{\mathbf m}^{\,\prime }}\rightarrow S_{{\mathbf m}}$ and decreases the probability through the reaction steps $S_{{\mathbf m}}\rightarrow S_{{\mathbf m}^{\,\prime }}$ where ${\mathbf m}^{\,\prime }$ is summed over all states from which $S_{{\mathbf m}}$ can be reached or vice versa. The two terms in the first line, for example, describe the two pseudoreactions modeling inflow and outflow of the material **A**, and further each reaction is represented by two steps. It is also worth noticing that stoichiometry requires two slightly different replication terms depending on whether the copy is correct or incorrect.

**Fig. 6:**
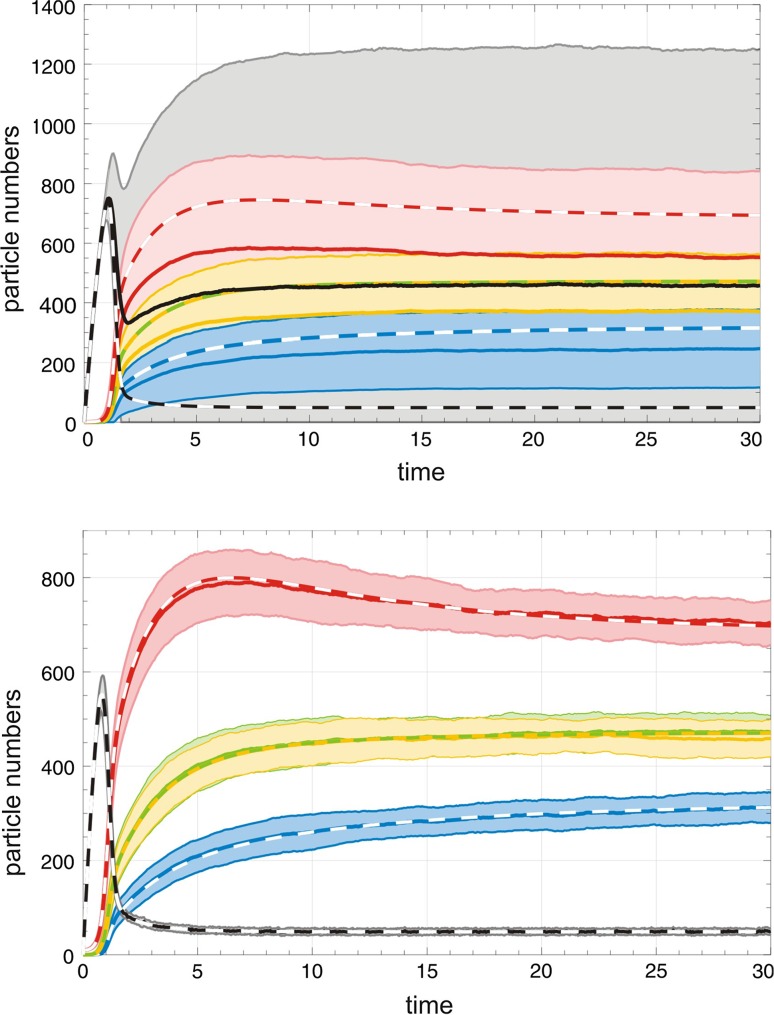
Quasispecies formation. The two plots show the formation of quasispecies from an initially empty reactor, $A(0)=0$, with replicators in seeding amounts. The system in the upper plot was initiated to be a single copy of the master sequence, $X_1(0)=1$ and $X_2(0)=X_3(0)=X_4(0)=0$. At the beginning the system might be extinguished by a single dilution event $X_1\rightarrow \varnothing$ and the high probability of extinction gives rise to enormously broad and overlapping one-standard-deviation bands. The initial values in the lower plot were chosen such that the probability of extinction is zero for all practical purposes: $X_1(0)=10$ and $X_2(0)=X_3(0)=X_4(0)=0$. Choice of parameters: $N=2000$; $k_1=0.011\,$[$M^{-1}$ $t^{-1}$], $k_2=k_3=0.010\,$[$M^{-1}$ $t^{-1}$], $k_4=0.009\,$[$M^{-1}$ $t^{-1}$], $r=0.5\,$[*V* *t*$^{-1}$], Color code: **A** black, ${{\mathsf{\mathbf X}}}_1$ red, ${{\mathsf{\mathbf X}}}_2$ yellow, ${{\mathsf{\mathbf X}}}_3$ green, and ${{\mathsf{\mathbf X}}}_4$ blue. Expectation values are shown as full lines, deterministic solutions as broken lines. Since $x_2(t)=x_3(t)$ is fulfilled for the parameter values used here the curve is shown as a yellow–green broken line

**Fig. 7:**
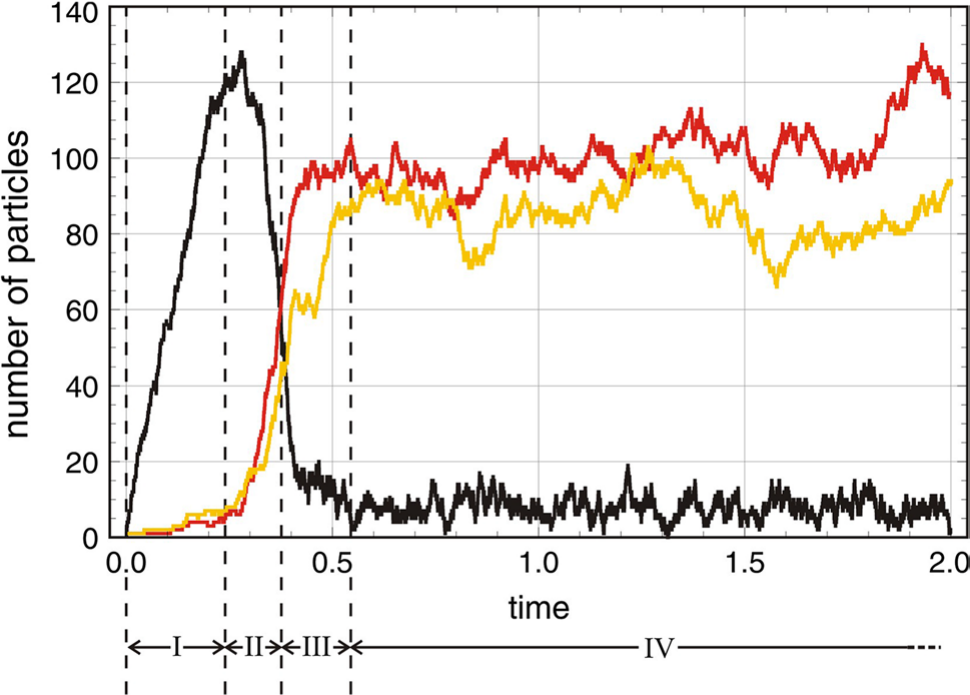
Sequence of phases in the approach towards a quasistationary state for $n=2$. A stochastic trajectory simulating competition and cooperation ("Competition and cooperation") of two species in the flow reactor is shown in the plot above. The corresponding master equation is derived from (14) by putting $\mathrm {Q}={\mathbb I}$. The stochastic process is assumed to start with an empty reactor except seeds for the two autocatalysts ${{\mathsf{\mathbf X}}}_1$ and ${{\mathsf{\mathbf X}}}_2$. It can be partitioned into four phases: (I) fast raise in the concentration of **A**, (II) a random phase where the decision is made into which final state—$S_0$, $S_1^{(1)}$, $S_1^{(2)}$ or $S_2$—the trajectory progresses, (III) the approach towards the final state—here $S_2$—and (IV) fluctuations around the values of the (quasi)stationary state. Comparison with the simple autocatalytic process in Fig. 4 reveals great similarity. Choice of parameter values: $k_1=0.099$, $k_2=0.101$ [M$^{-1}$t$^{-1}$], $l_1=0.0050$, $l_2=0.0045$ [M$^{-2}$t$^{-1}$], $a_0=200$, $r=4.0$ [V t$^{-1}$], pseudorandom number generator: *ExtendedCA, Mathematica*, seeds $s=631$. Initial conditions: $A(0)=0$, $X_1(0)=X_2(0)=1$. Color code: *A*(*t*) black, $X_1(t)$ red, and $X_2(t)$ yellow. The figure is adapted from Schuster (2017a)

Master equations are easily written down and stationary solutions can be derived by generally applicable techniques as it was shown for the flow equilibrium in "Deterministic and stochastic approaches" but explicit time-dependent solutions are very hard to obtain and known only in exceptionally simple cases [Schuster 2016e, pp 347–568]. Here, we shall use the simulation technique of sampling trajectories introduced already in "Autocatalysis in the batch reactor". Expectation values and second moments of variables can be computed through sampling of trajectories—an example is shown in Fig. 6—but often this approach exceeds the available computational facilities and it is necessary to interpret single trajectories. As examples we consider single trajectories in Fig. 4 and in Fig. 7. The process for convenience starting from an empty flow reactor is split into four phases: (i) establishment of the flow equilibrium of **A**, (ii) random decision on the (quasi)stationary state towards which the trajectory converges, (iii) the approach towards this (quasi)stationary state and (iv) fluctuations around the (quasi)stationary state. The separation into phases is made possible by the choice of suitable initial conditions.

### Competition, mutation and quasispecies

The bottom face of the three-dimensional parameter space— $\mathcal {A}$ in Fig. 5—is dealing with selection provided by the combination of competition and mutation. Natural selection at zero mutation rate leads to a homogeneous population of the fittest subspecies and at non-zero mutation rates this scenario yields selection of a fittest ensemble of subspecies, which has been characterized as quasispecies (Eigen and Schuster 1977; Domingo and Schuster 2016). Precisely, the quasispecies is the stable stationary long-time distribution of a population of subspecies undergoing replication and mutation: A population that consists of several genotypes present in time-dependent concentrations,

15 $$\begin{aligned} {\Uppi }(t)=x_1(t){{\mathsf{\mathbf X}}}_1\oplus x_2(t){{\mathsf{\mathbf X}}}_2\oplus \ldots \oplus x_n(t){{\mathsf{\mathbf X}}}_n \end{aligned}$$

has the stationary solution, $\lim _{t\rightarrow \infty }{\Uppi }(t)=\overline{\varvec{{\Upupsilon }}}=\overline{x}_1{{\mathsf{\mathbf X}}}_1\oplus \overline{x}_2{{\mathsf{\mathbf X}}}_2\oplus \ldots \oplus \overline{x}_n{{\mathsf{\mathbf X}}}_n$, which is called the *quasispecies* $\overline{\varvec{{\Upupsilon }}}$.

*Deterministic quasispecies* The deterministic or *continuous quasispecies* represents the unique deterministic long-time solution of the replication mutation problem, which is described in the flow reactor by the ODE (Schuster and Sigmund 1985):

16a $$\begin{aligned}\frac{\mathrm {d}{a}}{\mathrm {dt}}\ =\ -a\,\sum _{i=1}^n k_i x_i\,+\,r(a_0-a)\end{aligned}$$

16b $$\begin{aligned}\frac{\mathrm {d}{x_i}}{\mathrm {dt}}\ =\ a\sum _{j=1}^n Q_{ij}k_j\,x_j\,-\,r\,x_i\,,\ i=1,\ldots ,n\ . \end{aligned}$$

All early works on quasispecies dynamics were performed with the constraint of constant population size: $\sum _{i=1}^n x_i(t)=c=\mathrm {const}$ with $a(t)=a_0$ and $f_i=k_i a_0$ that gives rise to the differential equation

$$\begin{aligned} \frac{\mathrm {d}{x_i}}{\mathrm {dt}}\ =\ \sum _{j=1}^n Q_{ij}f_j\,x_j\,-\,\phi (t)\,x_i\,,\ \mathrm {with}\ \phi =\frac{\sum _{j=1}^n f_j x_j}{\sum _{j=1}^n x_j};\,i=1,\ldots ,n\,, \quad \quad \quad \mathrm{(16')} \end{aligned}$$

which fulfils ${\mathrm d}c/{\,\mathrm {dt}}=\sum _{i=1}^n{\mathrm d}x_i/{\,\mathrm {dt}}=0$.  Equations (16) and (16’) have identical solutions in normalized variables $\mathbf {\xi }_i(t)=x_i/\sum _{i=1}^n x_i(t)$ but the stability properties of the corresponding stochastic systems are different. [For more details see (Thompson 1974; Jones et al. 1976; Ebeling and Mahnke 1979; Jones and Leung 1981; Schuster and Sigmund 1985)].

The quasispecies as a function of the mutation rate parameter $\overline{\varvec{{\Upupsilon }}}(p)$ begins at $p=0$ as a homogeneous population containing exclusively the fittest subspecies ${{\mathsf{\mathbf X}}}_m$ and becomes a distribution of subspecies at nonzero *p*. This distribution consists of a most frequent sequence called master sequence, which is surrounded by a mutant cloud. Commonly, the most frequent or master sequence is also the fittest one, but this is not necessarily so: In case of strong stationary mutation flow from the mutant cloud to the master sequence, a less fit master may outgrow a fitter sequence with less efficient mutational backflow. At further increase in *p* the distribution broadens, and eventually ends up as the uniform distribution ${\mathcal U}\!:\,\{\widehat{x}_1=\widehat{x}_2=\cdots =\widehat{x}_n\}$ at $\widehat{p}=1-(\kappa -1)\widehat{p}/\kappa =1/\kappa$ where $n=\kappa ^l$ and $\widehat{x}_i=1/\kappa ^l\,(i=1,\ldots \kappa ^l)$ for sequences of chain length *l* over an alphabet with $\kappa$ letters.^6^ At the mutation rate $p=\widehat{p}$, correct and wrong digits are incorporated with the same frequency. The uniform distribution is the dynamical answer to the absence of any preference for nucleotide assignment: All subspecies in the stationary distribution occur with the same frequency. The quasispecies in the intermediate range is determined by the fitness landscape—the distribution of fitness values $f_i$ in sequence space, by the move set of allowed mutations as well as the mutation rate *p*. Typically, sharp transitions occur at some critical mutation rates $p=p_{\mathrm {tr}}$: The distribution changes smoothly from $p=0$ to $p=p_{\mathrm {tr}}$, where the distribution turns abruptly into another distribution. At the transition with the largest *p*-value $p=1/\kappa>p_{\mathrm {cr}}>p_{\mathrm {tr}}$, the quasispecies becomes an approximate uniform distribution. An explanation is straightforward: Above this critical transition, mutations occur too often to sustain sufficiently accurate reproduction of the template sequence and the result is *random replication*: In the long run, every sequence is obtained with the same probability. The transition has been characterized as *error threshold* (Eigen 1971; Eigen and Schuster 1977) since evolutionary dynamics does not sustain a structured long-time population at higher error rates, $p>p_{\mathrm {cr}}$. On typical fitness landscapes, the error threshold sharpens with increasing chain length *l* and becomes a first-order phase transition in the limit $l\rightarrow \infty$ (Tarazona 1992; Park et al. 2010; Huang et al. 2017).^7^

*Discrete quasispecies* In the continuous description, the quasispecies contains all species at finite positive concentrations no matter how small the concentrations might be. Dealing with less than one molecule per reactor volume, however, is unrealistic. Numbers of molecules are non-negative integers, $X_i\in \mathbb {N}$, subspecies distributions are truncated in reality and we call them stochastic or *discrete quasispecies*:

17 $$\begin{aligned} \widetilde{\varvec{{\Upupsilon }}}\ =\ \overline{X}_1{{\mathsf{\mathbf X}}}_1\oplus \overline{X}_2{{\mathsf{\mathbf X}}}_2\oplus \ldots \oplus \overline{X}_n{{\mathsf{\mathbf X}}}_n \ \ \mathrm {with}\ \ \overline{X}_i\,=\,{\left\{ \begin{array}{ll} \lfloor \overline{x}_i\rfloor \ &{}\mathrm {if}\ \overline{x}_i\ge 1\\ 0\ &{}\mathrm {if}\ \overline{x}_i<1\end{array}\right. }\ . \end{aligned}$$

The discreteness of the stochastic variables leads to a modification of the scenario for the mutation dependence of quasispecies $\overline{\varvec{{\Upupsilon }}}(p)$. In the mutation-free system, $p=0$, *survival of the fittest* is observed and the quasispecies consists of a single sequence: $\widetilde{\varvec{{\Upupsilon }}}=\overline{X}_m{{\mathsf{\mathbf X}}}_m=N{{\mathsf{\mathbf X}}}_m$ with *m* indicating maximal fitness, $f_m=\max \{f_i;i=1,\ldots ,n\}$, and *N* being the population size. At sufficiently small values of the mutation rate parameter *p*, no subspecies except the master exceeds the threshold $x_i\ge 1$ and the discrete quasispecies $\widetilde{\varvec{{\Upupsilon }}}$ still consists of a single fittest genotype ${{\mathsf{\mathbf X}}}_m$. The scenario in the small mutation rate regime—populations are almost always homogeneous except short non-stationary periods during which advantageous mutations take over the population—is tantamount to the strong selection–weak mutation scenario discussed in evolutionary biology (Gillespie 1983; Joyce et al. 2008).^8^ If the *p*-value is so large that for one or more subspecies $\overline{x}_i\ge 1$ is fulfilled, selection of a family of genotypes is observed: $\widetilde{\varvec{{\Upupsilon }}}$ consist of a master genotype ${{\mathsf{\mathbf X}}}_m$, together with a stationary distribution of sufficiently frequent mutants ${{\mathsf{\mathbf X}}}_i$ ($i\ne m$). The quasispecies becomes broader with increasing mutation parameter *p* until a threshold value $p_{\mathrm {cr}}$ is reached above which error propagation does not sustain a stationary state and the population ${\Uppi }$ drifts randomly through sequence space (Huynen et al. 1996; Higgs and Derrida 1991). Interestingly, the critical mutation rate $p_{\mathrm {cr}}$ can be derived from the continuous quasispecies theory.

As calculations of the time dependencies of the first two moments of the probability distribution of subspecies in the population, $P^{(\Uppi )}(t)$, reveal (Jones and Leung 1981)  (16’) is only marginally stable:

18a $$\begin{aligned} \frac{\mathrm {d}{\langle N\rangle }}{\mathrm {dt}}=\ \sum _{i=1}^n f_i\,\langle X_i\rangle \,-\,\langle \phi \bigl (\mathbf {X}(t)\bigr )\, N\rangle \ =\ 0\,,\end{aligned}$$

18b $$\begin{aligned} \frac{\mathrm {d}{\langle N^2\rangle }}{\mathrm {dt}}&=\ 2\sum _{i=1}^n f_i\langle X_i N\rangle \,-\,2\langle \phi \bigl (\mathbf {X}(t)\bigr )N^2\rangle \,+\nonumber \\&\,\,+\,2\sum _{i=1}^n f_i\langle X_i\rangle \,-\,\frac{\mathrm {d}{\langle N\rangle }}{\mathrm {dt}}\,=\,2\sum _{i=1}^n f_i\langle X_i\rangle \,,\ \ \mathrm {and}\end{aligned}$$

18c $$\begin{aligned} \mathrm {var}(N)=\ \langle N^2\rangle \,-\,\langle N\rangle ^2\ =\ 2\,\int _0^t\sum _{i=1}^n f_i\langle X_i(\tau )\rangle \,{\mathrm d}\tau \end{aligned}$$

The time derivative of the first moment vanishes as expected since the condition of a constant population size is fulfilled by the differential equation (16’). The variance, however, increases with time since the integrand in (18b) is always positive. After sufficiently long time, the fluctuation band becomes so large that the expectation value is irrelevant for the description of the system. The instability in Eigen’s quasispecies equation (Eigen 1971) is well known from similar problems: (i) the neutral birth-and-death process with equal birth and death parameters, $\lambda =\mu$, and (ii) the Wiener process. In both cases a constant expectation value is jeopardized by a variance that grows linearly with time. Stability against fluctuation is easily introduced into (16’): one needs only to give up the condition of strictly constant population size and to replace the denominator in $\phi (t)$ by a constant $\Uptheta$,

$$\begin{aligned} \phi (t)=\sum _{j=1}^n f_jx_j\bigm /\Uptheta \, , \end{aligned}$$

where $\Uptheta$ is the population size towards which the population converges after sufficiently long time. The approach chosen here, the implementation of a flow reactor as a physical device, yields a stable system as well.

Fluctuations at small particle numbers have different origins ("Autocatalysis in the batch reactor"): (i) conventional thermal fluctuations, (ii) enhanced fluctuations related to autocatalytic self-enhancement, and (iii) anomalous fluctuations in the stochastic variables arising from two or more quasistationary states. The standard deviations $\sigma (t)$ fulfil the $\sqrt{N}$-law for the resource **A** but are larger for the autocatalysts ${{\mathsf{\mathbf X}}}_1$, ${{\mathsf{\mathbf X}}}_2$, ${{\mathsf{\mathbf X}}}_3$, and ${{\mathsf{\mathbf X}}}_4$. The stationary states of the stochastic system are extinction $S_0$ and quasispecies $S_1$. Fig. 6 shows a typical example: The anomalous fluctuations in the upper plot are in full analogy to first-order autocatalysis (Fig. 4). Since the initial condition $X_1(0)=1$ was chosen, one outflow step may extinguish the population and the probability of dying out is indeed as large as 20%. The fluctuations bands are extremely broad, and large differences between the deterministic solution and the corresponding expectation values are observed. In the lower plot initial conditions $X_1(0)=10$ were chosen, which are sufficient to reduce the contributions of anomalous fluctuations practically to zero. Then, for a population size of $N=2000$ the concentration *a*(*t*) coincides with the expectation value $\mathrm {E}\bigl (A(t)\bigr )$ for all practical purposes and the fluctuations fall into a typical $\pm \sqrt{N}$-band. The autocatalysts, $X_1,\ldots ,X_4$, show broader than usual fluctuation bands because of self-enhancement as we saw in the intermediate range of the first-order autocatalytic reaction (Fig. 2). For long-times the standard deviation $\sigma (t)$ stays large in the quasispecies because the flow reactor is an open system and does not approach an equilibrium state (see also Fig. 4).

It is worth recalling what means stochasticity for quasispecies: (i) continuous concentrations are replaced by discrete particle numbers, (ii) fluctuations replace single line trajectories by bands within which trajectories follow a probability distribution, (iii) subspecies can be diluted out of the flow reactor and if this happens for all subspecies the population goes extinct giving rise to anomalous fluctuations, and (iv) error thresholds introduce random reproduction that is closely related to Motoo Kimura’s random drift. An increase in the error rate up to the error threshold leads to broadening of the mutant spectrum surrounding the master sequence. Above the thresholds, the populations migrate by random drift through sequence space.

### Competition and cooperation

The kinetic equations for replication describing the template induced, uncatalyzed and catalyzed processes are obtained from Eq. () by neglect of mutation. Then the kinetic differential equations for competition and cooperation of competitors result from (9) by setting $\mathrm {Q}={\mathbb I}$:

19a $$\begin{aligned} \frac{\mathrm {d}{a}}{\mathrm {dt}}\ = -a\,\sum _{i=1}^n\bigl (k_i\,+\,l_i x_{i+1}\bigr )\,x_i\ +\ r\,(a_0-a)\ \ \mathrm {and}\end{aligned}$$

19b $$\begin{aligned} \frac{\mathrm {d}{x_i}}{\mathrm {dt}}\ = \Bigl (a\bigl (k_i\,+\,l_i x_{i+1}\bigr )\,-\,r\Bigr )\,x_i\,; \ \ i=1\ldots ,n;\ i\!\!\!\!\mod n\,. \end{aligned}$$

Both equations contain terms of different molecularity in ${{\mathsf{\mathbf X}}}$ and this has the consequence that the dynamic behavior depends strongly on the total concentration $c=\sum _{i=1}^n x_i$, which in turn is determined by the amount of available resources, $a_0$: At sufficiently low concentration, the first-order terms, $k_i\,x_i$, dominate whereas the second-order terms, $l_i\,x_{i+1}x_i$ become important at high concentrations. No direct analytical solutions are available but exhaustive qualitative analysis is possible and the concentrations of the stationary solutions, $\overline{a},\,\overline{x}_i\,(i=1,\ldots ,n)$, can be computed analytically Schuster (2016a, d) (Table 2).

**Table 2:** Asymptotically stable stationary states of Eq. (19) with $n=3$Schuster (2016)

Symbol	Stationary values				Stability range
	$\overline{a}$	$\overline{x}_1$	$\overline{x}_2$	$\overline{x}_3$	
$S_0$	$a_0$	0	0	0	$0\le a_0\le \gamma _3$
$S_1^{(3)}$	$\gamma _3$	0	0	$a_0-\gamma _3$	$\gamma _3\le a_0\le \gamma _3+\gamma _{32}$
$S_2^{(1)}$	$\gamma _3$	0	$a_0-\gamma _3-\gamma _{32}$	$\gamma _{32}$	$\gamma _3+\gamma _{32}\le a_0\le \gamma _3+\gamma _{32}+\gamma _{31}$
$S_3$	$\alpha$	$\alpha _3$	$\alpha _1$	$\alpha _2$	$\gamma _3+\gamma _{32}+\gamma _{31}\le a_0$

The four stationary states are ordered with respect to increasing $a_0$-values of their asymptotically stable regime. The relations $k_1<k_2<k_3$ and $l_1>l_2>l_3$ between the rate parameters were assumed. The abbreviations $\gamma _3=r/k_3$, $\gamma _{31}=(k_3-k_1)/l_1)$, and $\gamma _{32}=(k_3-k_2)/l_2)$ are used for the combination of parameters. For the cooperative state $S_3$ the stationary concentration of **A** is obtained as one root of a quadratic equation (20) with two combinations of the rate parameters, $\psi =\sum _{i=1}^3 k_i/l_i$ and $\phi =\sum _{i=1}^3 1/l_i$, and $\alpha _i=(r-k_i\alpha )/(l_i\alpha )$ for $i=1,2,3$ and $\alpha =\overline{a}_1$ from  (20). The existence of the non-trivial stationary state requires a sufficiently small flow rate: $r\le (a_0+\psi )^2/4\phi$

The basis of the calculations of stationary states is the factorability of the r.h.s. of (19b). The relation $\overline{x}_i\cdot \bigl ((k_i+l_i\overline{x}_{i+1})\overline{a}-r\bigr )=0$ with $i\!\!\mod \!n$ is compatible with stationary states that fulfil either

$$\begin{aligned} \overline{x}_i^{(i)}=0 \, \mathrm{or} \, \overline{x}_i^{(ii)}=\frac{1}{ l_{i-1}}\left( \frac{r}{\overline{a}}-k_{i-1}\right) \,,\ \forall \,\,i=1,\ldots ,n\,, \end{aligned}$$

and the conservation condition $\overline{a}+\sum _{i=1}^n\overline{x}_i=a_0$. In total there are $2^n$ possible states out of which a single one is asymptotically stable for a given set of parameters. In Table 2 the results for $n=3$ are shown. The number of subspecies present at the stationary state, $n_{\mathrm {S}}$, is suitable for characterizing the state: $n_{\mathrm {S}}=0$ is the state of *extinction*, $n_{\mathrm {S}}=1$ is the state of *selection*, $n_{\mathrm {S}}=2$ is the state of *exclusion*, etc., and finally $n_{\mathrm {S}}=3=n$ occurs at the state of *cooperation* where all three subspecies are present. The numbers of long-time subspecies $n_{\mathrm {S}}$ depend on the resource input $a_0$. The relative size ordering of the parameters determines the identity of the selected, excluded, etc., subspecies. For the calculations shown in Table 2 the orderings $k_1<k_2<k_3$ and $l_1>l_2>l_3$ were applied and hence ${{\mathsf{\mathbf X}}}_3$ is selected and ${{\mathsf{\mathbf X}}}_1$ is excluded. Considering steady states as functions of the available resources, the state of *extinction* $S_0$ comes first at small $a_0$ and is stable for $a_0<r/k_3$, the state of *selection* of ${{\mathsf{\mathbf X}}}_3$, $S_1^{(3)}$, is stable in the range $r/k_3<a_0<r/k_3+(k_3-k_2)/l_2$, followed by *exclusion* of ${{\mathsf{\mathbf X}}}_1$, $S_2^{(1)}$, in the range $k_3+(k_3-k_2)/l_2<a_0<k_3+(k_3-k_2)/l_2+(k_3-k_1)/l_1$. Above this value for $a_0$ *cooperation* of all three subspecies is observed provided the flow rate is not too large: $r<r_{\mathrm {cr}}=(a_0+\psi )^2/4\phi$ with $\psi =\sum _{i=1}^3 k_i/l_i$ and $\phi =\sum _{i=1}^3 1/l_i$. The free concentration of **A** is obtained as solution of a quadratic equation^9^

20 $$\begin{aligned} \overline{a}_{1,2}\ =\ \frac{1}{2}\left( a_0+\psi \,\mp \,\sqrt{(a_0+\psi )^2-4r\phi }\right) , \end{aligned}$$

where the minus sign corresponds to the cooperative state $S_3$. The second solution belongs to an unstable state $S_3'$, which separates the basins of attraction of the states of cooperation and extinction. Above the critical flow rate, $r>r_{\mathrm {cr}}$ the states $S_3$ and $S_3'$ do not exist. For $n>3$, the situation becomes more complex since solutions may oscillate. Many systems with $n=4$ have oscillations with very weak damping factors, $n\ge 5$ commonly leads to undamped oscillations. The properties of these systems have been discussed extensively in previous publications to which we refer here (Schuster 2016e; Schuster and Sigmund 1985; Schuster 1978).

**Table 3:** Probabilities to reach quasistationary states in the cooperative regime with $n=2$ and different initial conditions

Initial values		Counted numbers of states in final outcomes			
$X_1(0)$	$X_2(0)$	$N_{S_0}$	$N_{S_1^{(1)}}$	$N_{S_1^{(2)}}$	$N_{S_2}$
1	1	$385.1\pm 23.6$	1481.0 ± 36.8	1719.6 ± 37.8	$6414.3\pm 53.8$
2	1	$77.4\pm 9.1$	1822.6 ± 41.6	367.6 ± 17.0	$7733.3\pm 38.3$
1	2	$71.6\pm 8.5$	280.6 ± 20.0	2075.8 ± 28.9	$7572.0\pm 39.2$
3	1	$15.0\pm 2.9$	1900.4 ± 30.9	74.69 ± 10.0	$8009.0\pm 35.3$
1	3	$14.0\pm 3.7$	53.1 ± 4.8	2180.5 ± 48.4	$7752.3\pm 53.8$
2	2	$14.9\pm 2.6$	303.7 ± 16.0	354.5 ± 23.8	$9326.8\pm 44.9$
3	3	0	70.2 ± 10.0	106.2 ± 10.9	$9823.4\pm 15.7$
4	4	0	12.1 ± 2.6	28.0 ± 5.0	$9959.9\pm 6.4$
5	5	0	2.5 ± 1.1	6.3 ± 2.6	$9991.2\pm 3.0$

The table provides probabilities of occurrence for all four possible long-term states: extinction $S_0$, selection of ${{\mathsf{\mathbf X}}}_1$ in $S_1^{(1)}$, selection of ${{\mathsf{\mathbf X}}}_2$ in $S_1^{(2)}$, or cooperation $S_2$. The counted numbers of events are sample means and unbiased standard deviations calculated from ten packages, each of them containing 10 000 trajectories computed with identical parameters and initial conditions, and differing only in the sequence of random events determined by the seeds of the pseudorandom number generator (*Extended CA, Mathematica*). Choice of parameters: $k_1=0.09\,$[M$^{-1}$t$^{-1}$], $k_2=0.11\,$[M$^{-1}$t$^{-1}$], $l_1=0.0011\,$[M$^{-2}$t$^{-1}$], $l_2=0.0009\,$[M$^{-2}$t$^{-1}$], $a_0=200$, $r=0.5\,$[V t$^{-1}$]. Initial value $A(0)=0$. Probabilities are obtained by multiplication by $10^{-4}$

Stochasticity has common effects on competition–cooperation systems like thermal fluctuations and fluctuation through autocatalytic enhancement. In addition, there are many more quasistationary states than the asymptotically stable states of the deterministic system. For example, states in which less efficient subspecies are selected show up as quasistationary states as well. In case of the smallest possible system with $n=2$ and $k_2>k_1$, we have four states: (i) the absorbing boundary as state of extinction $S_0$, (ii) the state of natural selection $S_1^{(2)}$ where the fittest variant ${{\mathsf{\mathbf X}}}_2$ is selected, (iii) the state of selection of the less fit subspecies ${{\mathsf{\mathbf X}}}_1$, and eventually (iv) the state of cooperation $S_2$. The relative stabilities of the individual states are reflected by the probabilities to reach these states by randomly chosen trajectories (Table 3). Parameters for the calculations shown in the table were chosen such that the corresponding deterministic system is situated in the cooperative domain in parameter space, and indeed the approach towards the cooperative state $S_2$ has always the largest probability. Again, initial particle numbers around $X_1(0)+X_2(0)=4$ are sufficient for strong dominance of the state corresponding to the deterministic solution. In case of $n=3$ we present expectation values of the four stochastic variables, *A*(*t*) and $X_i(t)$ ($i=1,2,3$) at some predefined time of the simulation end, $t_{\mathrm {end}}$ for different initial conditions and mutation rate parameters *p*. The values for $p=0.0$ refer to the pure competition–cooperation case discussed here (Table 4). As expected extinction plays a major role at small initial values, $X_i(0)=1,2,3$ ($i=1,2,3$), but $X_i(0)=4$ is already sufficient for coming close to the expectation values obtained for large initial values, $X_i(0)=10$ is enough for reaching the deterministic values for practical purposes.

In systems with $n\ge 4$ deterministic and stochastic solution curves oscillate. The solutions of the ODE’s are different for $n=4$ where weakly damped oscillations occur and for $n\ge 5$ showing undamped *relaxation oscillations* (Phillipson et al. 1984). In the stochastic approach, the systems die out after population numbers of individual subspecies went beyond $X(t)=1$, and for sufficiently large population sizes, four-membered systems may survive for very long time whereas systems with five or more members go extinct. Cases with $n=5$ are well suited for studying the transition from selection to cooperative dynamics through increase of the parameter ration ratio $h/f=l/k$ (Fig. 9). At dominant competition or small *h*-values the system approaches selection of the fittest as long-time solution. The upcoming role of cooperation in a series of systems with increasing parameters *l* can be nicely illustrated by a series of plots of trajectories from selection to somewhat chaotic looking intermediate scenarios and further to oscillatory hypercycle dynamics (see Fig. 9 where a nonzero mutation rate parameter *p* was chosen and where accordingly quasispecies formation instead of selection is observed).

**Table 4:** Long-time behavior in the cooperation-mutation system with $n=3$ and different initial conditions

Mutation	Initial values			Expectation values at $t=30$				Counts
*p*	$X_1(0)$	$X_2(0)$	$X_3(0)$	*E*(*A*)	$E(X_1)$	$E(X_2)$	$E(X_3)$	$P(S_3)$
0	1	1	1	279.3	44.56	36.06	39.58	0.301
0	2	2	2	81.53	117.7	94.9	105.4	0.814
0	4	4	4	2.023	146.1	119.6	132.2	0.996
0	10	10	10	0.376	147.0	120.0	132.6	1.0
0	Deterministic			0.377	147.0	120.3	132.3	1
0.05	1	1	1	177.2	81.66	68.16	72.64	0.432
0.05	2	2	2	28.57	136.1	113.8	121.5	0.937
0.05	4	4	4	0.383	147.0	122.2	130.4	1.0
0.05	Deterministic			0.377	146.7	122.3	130.5	1
0.1	1	1	1	161.6	86.95	74.39	76.96	0.599
0.1	2	2	2	28.57	136.1	113.8	121.5	0.957
0.1	4	4	4	0.383	147.0	122.2	130.4	1.0
0.1	Deterministic			0.377	146.7	122.3	130.5	1

The table provides expectation values at the time of the end of the simulation ($t_{\mathrm {end}}=30$) for different mutation rate parameters *p* and different initial conditions. Choice of parameters: $l_1=0.011$, $l_2=0.010$, $l_3=0.009\,$[M$^{-2}$t$^{-1}$], $a_0=400$, $r=0.5\,$[V t$^{-1}$]. Initial value $A(0)=0$

**Fig. 8:**
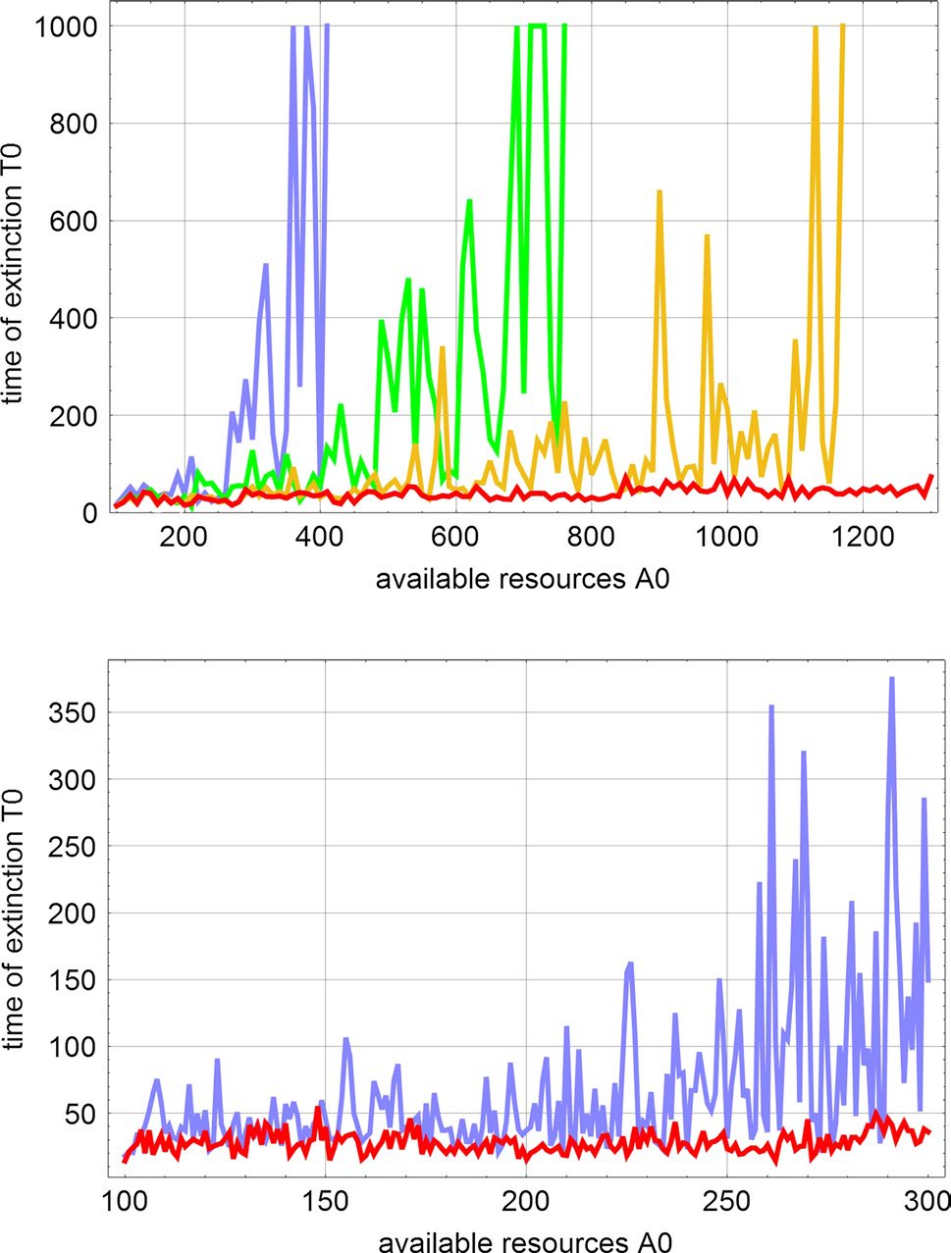
Times to extinction as a function of available resources in the five membered cooperative system ($$n=5$$). Extinction times $$T_0$$ of the populations $${\Uppi }$$ are shown for different amounts of available resources measured as inflow concentrations $$a_0$$ or $$A_0$$ when expressed in numbers of molecules per unit volume. The upper diagram presents the data at a resolution of ten molecules ($$\Delta A_0=10$$; 100, 110, 120,$$\ldots$$) for four different values of the mutation rate parameter: $$p=0.0000$$ (red), $$p=0.0005$$ (yellow), $$p=0.0010$$ (green), and $$p=0.0020$$ (blue). $$T_0$$-values above 1000 are truncated at this value. The lower diagram shows the two plots $$\bigl (p=0.0000$$ (red) and $$p=0.0020$$ (blue)$$\bigr )$$ at the highest possible resolution ($$\Delta A_0=1$$). Choice of other parameters: $$r=0.5\,$$[$$V^{-1}$$$$t^{-1}$$], $$l_1=l_2=l_3=l_4=l_5=0.01\,$$[$$M^{-2}$$$$t^{-1}$$]. Pseudorandom number generator: *Extended CA, Mathematica*, seed: $$s=491$$. Initial conditions: $$A(0)=0$$, $$X_1(0)=X_2(0)=X_3(0)=X_4(0)=X_5(0)=5$$

### Cooperation and mutation

The combination of cooperation and mutation reveals a less common role of mutation in addition to the creation of diversity through variation. In principle, mutation can reintroduce extinguished subspecies into the population. Here, we shall focus on this aspect and, in particular, study the influence of the mutation rate parameter *p* on extinction times. To study the role of mutation in low-dimensional cooperative systems ($n=2,3$) expectation values of the stochastic variables *A*(*t*), $X_1(t)$ and $X_2(t)$, or $X_2(t)$ and $X_3(t)$ were calculated at predefined times ($t_{\mathrm {end}}$) and compared with the probabilities of trajectories to end up in the cooperative state, $S_2$ or $S_3$, respectively. For the initial conditions $X_1(0)=X_2(0)\bigl (=X_3(0)\bigr )>4$ the results are practically indistinguishable from the deterministic values. An increase in the mutation rate parameter *p* shows the expected influence: Extinguished subspecies can be reintroduced and this increases the probability of reaching the cooperative state, $S_2$ or $S_3$. The case $n=3$ is shown as an example in Table 4.

The oscillating systems are more difficult to investigate. Here, we consider the time of extinction of the entire population as a function of the mutation rate and the available resources, $T_0(p,A_0)$. The results are shown in Fig 8: The extinction times $T_0$ show very strong scatter and their appearance is dependent on the resolution of the calculations. By resolution, we mean here the number of molecules **A** in $A_0$ between two neighboring points. The highest resolution is achieved when the calculations are performed with every (integer) number of $A_0$ molecules, e.g., $\Delta A_0=1$ yields 100, 101, 102, $\ldots$ . Computations at somewhat lower resolutions are less time consuming and provide in essence the same results. The plots shown in Fig. 8 show enormous scatter but, nevertheless, allow for drawing two conclusions: (i) In the mutation-free case the extinction time $T_0$ is independent of the amount of available resources up to a value $A_0\approx 1300$, and (ii) for non-zero mutation rates a kind of noisy or stochastic threshold phenomenon is observed. Considering the noisy function $T_0(p,A_0)$ and taking $A_0$ at the first value of $T_0(p,A_0)\ge 1000$ we find for the parameter values applied in Fig. 8: $A_0^{(T_0\ge 1000)}=A_0^{(\mathrm {cr})}=1130, 690, 360$ for the mutation rates $p=0.0005, 0.0010, 0.0020$, respectively. As expected the threshold moves to lower $A_0^{(\mathrm {cr})}$-values with increasing mutation rate. The behavior of the extinction times $T_0(A_0)$ is similar for $n=4$ but the critical concentrations $A_0^{(\mathrm {cr})}$ for the different *p*-values lie much closer together and the analysis is more difficult. Considering survival at constant resources $A_0$ reveals a *mutation threshold* above which the population survives to long times.

Hypercycle extinction is an example that reflects well the expected increase in lifetime with increasing mutation rate. One general remark nevertheless is important: This mechanism of reintroduction of extinguished subspecies requires that template and mutant are close relatives and that the Hamming distance between them is not too large. What we need in reality, however, is not a perfect revertant being genetically identical to the lost original, we need only a subspecies that can replace the original with respect to its phenotypic function. Suppression of deleterious mutations (Gorini and Beckwith 1966; Hartman and Roth 1973; Prelich 1999) as well as the relation between protein sequence, structure and functional efficiency (Albery and Knowles 1976, 1977) have been extensively studied in the last decades of the twentieth century.

### The complete model

Completion of the model brings together the three faces of the coordinate system in Fig. 5 and is concerned with an analysis of the dynamics in the interior. An appropriate strategy for analyzing the interior consists in choosing certain type of behavior on one of the three faces and increasing the third parameter from zero to the value of interest. Raising the third parameter will change the dynamic behavior either gradually or in threshold-like manner or stepwise through a cascade of bifurcations. Illustrative prototype examples are seen through rising the mutation rate in competitive or cooperative reproduction, or with the introduction of cooperation into Darwinian systems.

**Fig. 9:**
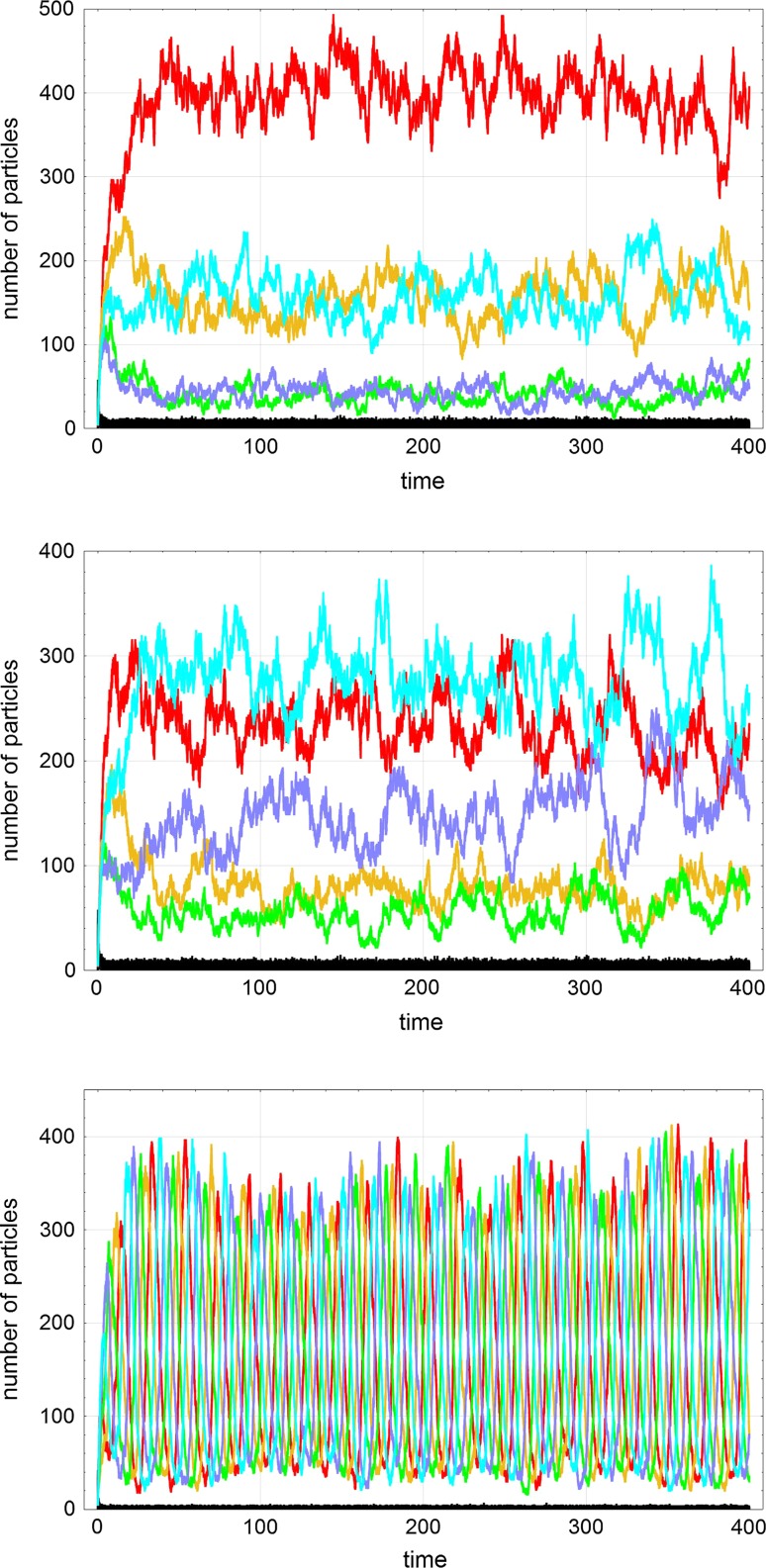
The transitions from competition to cooperation in the system with $$n=5$$ The transitions from competition to cooperation in the system with $$n=5$$. The three plots show single trajectories for three different scenarios in the flow reactor: (i, topmost plot) the quasispecies scenario with a dominant master sequence ($${{\mathsf{\mathbf X}}}_1$$), (ii, middle plot) an intermediate scenario with irregular dynamics and two dominating species ($${{\mathsf{\mathbf X}}}_1$$ and $${{\mathsf{\mathbf X}}}_5$$), and (iii, bottom plot) the stochastic hypercycle scenario with irregular, undamped oscillations. Choice of parameter: $$k_1=0.150$$, $$k_2=k_5=0.125$$, $$k_3=k_4=0.100$$ [$$M^{-1}$$$$t^{-1}$$], $$l_1=l_2=l_3=l_4=l_5=l$$, $$l=0.0$$ (topmost plot), $$l=0.002$$ (middle plot), $$l=0.01$$ [M$$^{-2}$$t$$^{-1}$$] (bottom plot), $$a_0=800$$, $$r=0.5$$ [V t$$^{-1}$$], $$p=0.075$$, pseudorandom number generator: *ExtendedCA, Mathematica*, seeds $$s=491$$. Initial conditions: $$A(0)=0$$, $$X_1(0)=X_2(0)=X_3(0)=X_4(0)=X_5(0)=5$$. Color code: $$\mathsf{A}(t)$$ black, $$\mathsf{X}_1(t)$$ red, $$\mathsf{X}_2(t)$$ yellow, $$\mathsf{X}_2(t)$$ green, $$\mathsf{X}_2(t)$$ blue, and $$\mathsf{X}_5(t)$$ cyan

The characteristic dependence of the population dynamics on *n*, the number of subspecies, prevails also in the full model. For small numbers ($n=2,3$) and $p=0$ the transition from the competitive to the cooperative system has been discussed in "Competition and cooperation". An increase of the cooperation parameter $h=l\,A(t)$ leads in steps from selection of the fittest to a cooperative state with all subspecies present. Oscillating systems ($n\ge 4$) are more spectacular since the hypercycles are unstable at $p=0$ in the stochastic approach and raising the cooperation parameter *h* leads from selection to extinction. In the intermediate parameter range where the deterministic system shows stepwise increase in the number of coexisting subspecies ($1,2,\ldots ,n$ or, expressed phenomenologically, selection, exclusion, $\ldots$, cooperation) the stochastic approach yields highly irregular dynamics with different numbers of non-extinguished subspecies whereby the number of species present increases with increasing values of the cooperation parameter *h*. The scenario in parameter space is completed by considering the series of states at different mutation rate parameters *p*. The case $n=5$, which for large *h*-values leads to undamped oscillations with a stochastic contribution to the amplitude, was chosen to facilitate the illustration (Fig. 9): At zero mutation rate $p=0$ the series with increasing *h* is selection $\rightarrow$ irregular dynamics with two species $\rightarrow$ irregular dynamics with three species $\rightarrow \ldots \rightarrow$ extinction. At intermediate *p*-values, we find quasispecies $\rightarrow$ irregular dynamics $\rightarrow$ oscillations with highly irregular spacings. At high mutation rates, we are dealing with quasispecies $\rightarrow$ irregular dynamics with increasing numbers of dominant subspecies $\rightarrow$ stochastic hypercycle dynamics (Fig. 9).

## Concluding remarks

The model presented here has been conceived with modular structure in the sense that different mechanisms can be applied for each of the three basic components. Here, it has been presented in its simplest form. Each of the three modules, competition, cooperation and variation, can be made arbitrarily complex. Variation, for example, can be extended to include more elaborate mutation mechanisms and recombination as well as environmental influences. Even in case of viruses the reproduction mechanism is commonly much more elaborate and comprises a whole molecular machinery instead of a single enzyme. Virus reproduction may include also the defense system of the host, epigenetic phenomena could be taken into account through simultaneous consideration of several generations, and for higher organisms the real challenge in reproduction is to deal with the enormous complexity of development in a form that is simple enough for modeling. Cooperation at the molecular level could also involve reproductive autocatalytic networks whereas social phenomena in reproductive groups or societies represent the currently highest step in the open ended complexity increase of biological evolution. Cooperation has been frequently modeled by game theory Maynard Smith (1982); Hofbauer and Sigmund (1998). There is no limitation to make the model more complex, the problem evidently is to include the desired phenomena but to keep the model sufficiently simple for mathematical analysis or simulation.

In the simple form in which the model was introduced here, it has been tested experimentally by *in vitro* evolution experiments [For an overview of early works on this subject see (Spiegelman 1971; Biebricher 1983); as a recent review we mention (Joyce 2007)]. The kinetic equations describing replication and mutation were introduced 1971 by Manfred Eigen in his scholarly written paper on self-organization of biological macromolecules (Eigen 1971). Eigen’s mutation-selection equation describes the evolution of the distribution of asexually reproducing genotypes in a population of constant size *N*. Correct replication and mutation are seen as parallel chemical reactions leading to a uniquely defined stationary population called *quasispecies* (Eigen and Schuster 1977). RNA replication catalyzed by single virus specific enzymes from RNA bacteriophages provides a bridge from chemistry to biology: The mechanism of the replication process is well understood in all molecular details (Biebricher 1983; Biebricher et al. 1984, 1985) and an appropriate replication assay serves for in vitro evolution studies (Mills et al. 1967; Biebricher 1983). The mutation-selection scenario was found to provide an appropriate molecular basis for understanding also virus evolution [For a recent survey see the contributed volume (Domingo and Schuster 2016)]. More complex systems, for example, bacteria and populations of cancer cells, were found to be describable by quasispecies theory as well (Bertels et al. 2017; Covacci and Rappuoli 1998; Napoletani et al. 2013; Brumer et al. 2006).

In the *strong mutation scenario*$^8$ Darwin’s view of evolution has to be modified. Not a single fittest genotype is selected but a uniquely defined distribution of genotypes, which is represented by the largest eigenvector of a value matrix that represents the long time or stationary solution of Eigen’s mutation-selection equation. The mean fitness of populations is not always optimized since situations can be constructed in which the fitness is decreasing in the approach towards the stationary state. A trivial but illustrative example of decreasing fitness during evolution considers a homogeneous population consisting exclusively of fittest genotypes as initial condition: Mutations introduce mutants into the population and since they have lower fitness by definition the mean fitness is doomed to decrease. Such situations, however, are rather rare and Darwinian optimization still remains a very powerful heuristic that applies to almost all scenarios. For error rate parameters exceeding a critical value $p_{\mathrm {cr}}$, the largest eigenvector approaches the uniform distribution over the entire sequence space, which is the exact solution for the value $p=\widehat{p}$ leading to incorporations of correct and incorrect nucleotides with equal probabilities—for binary sequences this happens at $\widehat{p}=1-\widehat{p}=\frac{\text {{1}}}{\text {{2}}}$. In realistic populations, the uniform distribution is incompatible with a discrete quasispecies (17). Instead populations are observed that migrate randomly through sequence space Higgs and Derrida (1991); Huynen et al. (1996).

In the second half of the twentieth century, most of the molecular insights into reproduction and inheritance came from viruses and bacteria and a high percentage of molecular biologists thought that the basic regulation mechanisms of gene activities are understood. Eukaryotic cells, however, are not “giant bacteria”. Although the genetic code is the same, the gene expression and inheritance system of higher organisms are different from the prokaryotic one and much more complex. A true wealth of information on eukaryotic cells has been discovered in the past fifty years, but gene expression in animals, plants, and fungi is still a subject of current cutting-edge research. Most of the recently revealed gene expression regulation mechanisms are subsumed under the notion of *epigenetics* for which an operational definition has been proposed at the Cold Spring Harbor Meeting in the year 2008 (Berger et al. 2009):

*An epigenetic trait is a stably heritable phenotype resulting from changes in a chromosome without alterations in the DNA sequence.*

The diversity of epigenetic effects on gene regulation is enormous. It ranges from specific methylation of DNA, in particular cytosine methylation in position 5 of CpG elements (Zemach et al. 2010), histone methylation and acetylation (Lawrence et al. 2016) to post-transcriptional RNA-methylation of adenine in position 6 (Barbosa Dogini et al. 2014; Yue et al. 2015) and small interfering RNAs (He and Hanon 2004). Epigenetics provides an extremely diverse, complex and flexible richness of regulatory actions on genes, which so far was not yet cast into a comprehensive theory and precisely this is one of the greatest challenges for the future of evolutionary biology.

There is neither a convincing theoretical model nor experimental evidence that Darwinian evolution leads to an obligatory increase in complexity. The combination of competitive selection and cooperation, however, may lead from one level of complexity to the next higher one by integration of competitors through cooperation (Szathmáry and Maynard Smith 1995; Schuster 1996). The evolution model presented here proposes a mechanism for this integration of competitors and identifies the abundance of resources as one driving force towards higher complexity. This simple model distinguishes four steps (Schuster 1996): (i) Initially the systems consists of independent replicators competing for a single resource, (ii) the capability of cooperative interaction allows to form an autocatalytic network, which couples the replicators and suppresses competition but makes the network vulnerable to exploitation by parasites, which consume resources without contributing a share to the common properties, (iii) the members of the autocatalytic network are separated from the environment by means if a suitable boundary that prevents the system from exploitation and allows for the formation of a new unit at a hierarchically higher level, and (iv) the individual units at the higher level diversify by variation, compete for common resources, Darwinian selection sets in and takes place now a the higher level. The previously autonomous units at the lower level lost their autonomy at least in part when they were integrated into the higher unit of selection. Although modeling major transitions as shown in "Competition and cooperation" is not difficult, suitable experimental molecular models are very hard to conceive, because second- and higher-order autocatalytic systems consist almost always of complex reaction networks rather than single-step reactions [as examples for attempts to construct simple systems of this kind see (McCaskill 1997; Wlotzka and McCaskill 1997)]. John Maynard Smith and Eörs Szathmáry collected a true wealth of evidence for the historic occurrence of such major evolutionary transitions (Maynard Smith and Szathmáry 1995) in the evolution of life.

It is illustrative to think about transitions in terms of thresholds: Up to a certain value of the transition determining parameter the typical feature is hardly detectable and does not become evident before the parameter exceeds the transitions value. Accordingly, such thresholds correspond to sharp transitions. Nevertheless, it appears useful to be less fussy and to accept the notion of threshold also for smooth transitions. On the three faces of the coordinate system (Fig. 5) we observe an error threshold between selection and random replication, a cooperation threshold between selection and symbiosis, and a mutation threshold that separates the regime of independent replication of all subspecies from mutual support through frequent mutation.

Understanding evolution implies knowledge on the relation between genotypes being DNA or RNA sequences and phenotypes giving rise to all fitness relevant parameters. The metaphor of an abstract fitness landscape has been originally introduced by Sewall Wright for the purpose of illustration (Wright 1922). In formal mathematical terms such a relation can be modeled as a mapping from a genotype or sequence space into fitness values. In molecular structural biology such a mapping is split into two parts: (i) a mapping from sequences into structures or genotypes into phenotypes, and (ii) a second mapping assigning fitness values to structures or phenotypes (Schuster 2016). Computer models of RNA evolution with sequence–secondary structure–fitness mappings have been studied in the past (Fontana and Schuster 1987; Fontana et al. 1989; Fontana and Schuster 1998a, b and these studies provided the basis for a definition of evolutionary nearness of phenotypes in the presence of neutrality (Stadler et al. 2001). With more and more data on sequences and fitness values of mutants becoming currently available fitness landscapes may also be determined directly by experiment (Kouyos et al. 2012) and it is not risky to predict that genotype–phenotype relations will become an integral part of evolution models in the near future. Then evolution can be described in a self-contained manner where the genotype–phenotype mapping is a genuine part of the evolving system.

## Footnotes

1 The term *linear* reaction kinetics is used in this contribution for reactions and flow terms of zero and first order, which give rise to constants or linear functions in the kinetic ODEs. First-order kinetics gives rise to exponential functions, which become linear in $\ln [\mathsf{X}]/t$-plots.

2 A stochastic quasistationary state is a state towards which the system converges stochastically in the long-time limit and around which it fluctuates. It is not an absorbing boundary, and if true asymptotically stable stationary states exist the system converges to one of them in the limit $t\rightarrow \infty$ although the mean time of convergence may be extremely long.

3 We shall use different letters for the rate parameters: For $m=0$ we use $g\equiv h$, for $m=1$ $g\equiv k$, and for $m=2$ $g\equiv l$. It is worth recalling the different dimensions: *h* [t$^{-1}$], *k* [M$^{-1}$t$^{-1}$], and *l* [M$^{-2}$t$^{-1}$] that are the same in both directions.

4 Here the expression $(x)_n=x!/(x-n)!$ denotes the falling Pochhammer symbol.

5 We remark that the deterministic kinetic Eqs. for (8b) and (8c) were extensively studied under the simplifying assumption of *constant population size* $\sum _{i=1}^n x_i(t)=c_0=\mathrm {const}$ (Eigen 1971; Eigen and Schuster 1978; Eigen and McCaskill 1989). The deterministic solution curves formulated in relative concentrations $\upxi _i(t)=x_i(t)\!\bigm /\!\sum _{i=1}^n x_i(t)$ are identical for the CSTR and for constant population size (Schuster and Sigmund 1985). As we mentioned already in "Idealized environments" the stochastic system is unstable for unregulated constant population size (Jones and Leung 1981) and a proper description has to account explicitly for the physical setup applied, here the flow reactor.

6 The value $\kappa =2$ is used here and applies for binary sequences. For the natural AUGC-nucleotide alphabet we have $\kappa =4$.

7 We mention that some model landscapes like the additive landscape or the multiplicative landscape sustain smooth rather than sharp transitions (Wiehe 1997). For a recent update and a review of the state of the art in the relation between fitness landscape and selection dynamics see (Schuster 2016d).

8 Biologists (Dean and Thronton 2007; Sniegowski and Gerrish 2010) and computer scientists commonly distinguish strong and weak mutation. The weak mutation scenario assumes that adaptive mutations are sufficiently rare and do not interfere with the selection process but initiate replacements of currently fittest genotypes by still fitter variants. The strong mutation scenario is characterized by sufficiently large values of *p* that give rise to the quasispecies dynamics described here [for details see (Domingo and Schuster 2016)] and to mutation induced cooperative dynamics ("Cooperation and mutation").

9 It is worth mentioning that Eq. (20) and the solutions for $\overline{a}_{1,2}$ are valid for arbitrary *n* provided the summations in $\psi$ and $\phi$ are adjusted accordingly.
